# Mutagenicity of carcinogenic heterocyclic amines in *Salmonella typhimurium* YG strains and transgenic rodents including *gpt* delta

**DOI:** 10.1186/s41021-021-00207-0

**Published:** 2021-09-16

**Authors:** Takehiko Nohmi, Masahiko Watanabe

**Affiliations:** 1 Division of Pathology, National Institute of Health Sciences, 3-25-26 Tonomachi, Kawasaki-ku, Kawasaki-shi, Kanagawa 210-9501 Japan; 2 School of Pharmacy, Shujitsu University, 1-6-1 Nishigawara, Naka-ku, Okayama, 703-8516 Japan

**Keywords:** Heterocyclic amines, Mutagenicity, Ames test, *Salmonella typhimurium* YG strains, Acetyltransferase, Transgenic, *gpt* delta, Carcinogenicity

## Abstract

Chemical carcinogens to humans have been usually identified by epidemiological studies on the relationships between occupational or environmental exposure to the agents and specific cancer induction. In contrast, carcinogenic heterocyclic amines were identified under the principle that mutagens in bacterial in the Ames test are possible human carcinogens. In the 1970s to 1990s, more than 10 heterocyclic amines were isolated from pyrolysates of amino acids, proteins, meat or fish as mutagens in the Ames test, and they were demonstrated as carcinogens in rodents. In the 1980s and 1990s, we have developed derivatives of the Ames tester strains that overexpressed acetyltransferase of *Salmonella typhimurium*. These strains such as *Salmonella typhimurium* YG1024 exhibited a high sensitivity to the mutagenicity of the carcinogenic heterocyclic amines. Because of the high sensitivity, YG1024 and other YG strains were used for various purposes, e.g., identification of novel heterocyclic amines, mechanisms of metabolic activation, comparison of mutagenic potencies of various heterocyclic amines, and the co-mutagenic effects. In the 1990s and 2000s, we developed transgenic mice and rats for the detection of mutagenicity of chemicals in vivo. The transgenics were generated by the introduction of reporter genes for mutations into fertilized eggs of mice and rats. We named the transgenics as *gpt* delta because the *gpt* gene of *Escherichia coli* was used for detection of point mutations such as base substitutions and frameshifts and the *red/gam* genes of λ phage were employed to detect deletion mutations. The transgenic rodents *gpt* delta and other transgenics with *lacI* or *lacZ* as reporter genes have been utilized for characterization of mutagenicity of heterocyclic amines in vivo. In this review, we summarized the in vitro mutagenicity of heterocyclic amines in *Salmonella typhimurium* YG strains and the in vivo mutagenicity in transgenic rodents. We discussed the relationships between in vitro and in vivo mutagenicity of the heterocyclic amines and their relations to the carcinogenicity.

**Supplementary Information:**

The online version contains supplementary material available at 10.1186/s41021-021-00207-0.

## Background

International Agency for Research on Cancer (IARC) has listed more than 100 agents that are carcinogenic to humans (Group 1) [1]. These carcinogenic agents were identified by epidemiological studies on the relationships between specific cancer induction and occupational or environmental exposure to these agents. For example, *o*-toluidine has been recognized as a human carcinogen because of the bladder cancer of workers in dye industries [2]. Asbestos has been identified as a human carcinogen because of its strong association with mesothelioma and lung cancer in construction and factory workers [3]. 1,2-Dichloropropane was included in Group 1 agents because of bile duct cancer in employees in the printing industry [4]. Exposure to vinyl chloride monomers induces angiosarcoma in the liver of industrial workers [5]. In this regard, the discovery of carcinogenic heterocyclic amines is unique because they were initially identified as mutagens in bacteria in the Ames test and then demonstrated as carcinogens in rodents [6]. 3-Amino-1,4-dimethyl-5*H*-pyrido[4,3-*b*]indole (Trp-P-1), 3-amino-1-methyl-5*H*-pyrido[4,3-*b*]indole (Trp-P-2), 2-amino-6-methyldipyrido[1,2-*a*:3’,2’-*d*]imidazole (Glu-P-1), 2-aminodipyrido[1,2-*a*:3’,2’-*d*]imidazole (Glu-P-2), 2-amino-9*H*-pyrido[2,3-*b*]indole (AαC) and 2-amino-3-methyl-9*H*-pyrido[2,3-*b*]indole (MeAαC) were isolated from the pyrolysates of amino acids and proteins as potent mutagens in *Salmonella enterica* subsp. *enterica* serovar Typhimurium (*Salmonella typhimurium*) TA98 (Table 1) (Supplementary Fig. 1 and Table 1) [7–9]. 2-Amino-3-methylimidazo[4,5-*f*]quinoline (IQ), 2-amino-3,4-dimethylimidazo[4,5-*f*]quinoline (MeIQ), 2-amino-3,8-dimethylimidazo[4,5-*f*]quinoxaline (MeIQx) and 2-amino-1-methyl-6-phenylimidazo[4,5-*b*]pyridine (PhIP) were isolated from broiled meat or fish also as potent mutagens in strain TA98 [10–14]. Fortunately, there is no accidental excess exposure to heterocyclic amines in humans. Therefore, the link between the consumption of heterocyclic amines and human cancer is still debatable. However, IQ is ranked as a probable human carcinogen (Group 2A), and others are ranked as possible human carcinogens (Group 2B) by IARC [15]. Therefore, the history of research on carcinogenic heterocyclic amines would provide valuable lessons about the roles of *in vitro* and *in vivo* mutagenicity assays in the discovery of human carcinogens. In this review, we have first summarized *in vitro* mutagenicity of heterocyclic amines in *Salmonella typhimurium* YG strains that are highly sensitive to the mutagenicity of aromatic amines and nitro aromatics [16]. Second, we have presented the review of *in vivo* mutagenicity of heterocyclic amines in transgenic rodents such as *gpt* delta mice/rats, *lacI* mice/rats and *lacZ* mice [17, 18]. Finally, we have discussed the effectiveness and limitations of the mutagenicity assays to discover human carcinogens and the cancer risk of heterocyclic amines in daily life.

**Table 1:** *Salmonella typhimurium*^1^ YG strains

Strain	Description	Reference
TA98	TA1538 (pKM101)	[19, 20]
TA98NR	as TA98 but deficient in classical nitroreductase	[21]
TA98/1,8-DNP_6_	as TA98 but deficient in *O*-acetyltransferase	[21]
TA1538/NR	as TA1538 but deficient in classical nitroreductase	[22]
TA1538/1,8-DNP	as TA1538 but deficient in *O*-acetyltransferase	[22]
YG1006	TA1538/1,8-DNP (pYG121)^2^	[16]
YG1012	TA1538/1,8-DNP (pYG213)^3^	[23]
YG1019	TA1538/1,8-DNP (pYG219)^4^	[23]
YG1020	TA98 (pBR322-AP^s^)	[16]
YG1021	TA98 (pYG216)^5^: a nitroreductase-overproducing strain	[24]
YG1024	TA98 (pYG219)^4^: an *O*-acetyltransferase-overproducing strain	[16]
YG1024NR	TA98NR (pYG219)^4^: as TA98NR (nitroreductase-deficient) but *O*-acetyltransferase is overexpressed	[25]
YG1041	TA98 (pYG233)^6:^ a nitroreductase and *O*-acetyltransferase-overproducing strain	[26]
TA100	TA1535 (pKM101)	[19, 20]
TA100NR	as TA100 but deficient in classical nitroreductase	[21]
TA100/1,8-DNP	as TA100 but deficient in *O*-acetyltransferase	[21]
YG1025	TA100 (pBR322-AP^s^)	[16]
YG1026	TA100 (pYG216)^5^: a nitroreductase-overproducing strain	[24]
YG1029	TA100 (pYG219)^4^: an *O*-acetyltransferase-overproducing strain	[16]
YG1042	TA100 (pYG233)^6^: a nitroreductase and *O*-acetyltransferase-overproducing strain	[26]

^1^The formal name *Salmonella enterica* subsp.*enterica* serovar Typhimurium is abbreviated as *Salmonella typhimurium* in Tables, Figures and the text

^2^Plasmid pYG121 is a derivative of plasmid pBR322 having part of the chromosome of TA1538 including the *oat* gene encoding *O*-acetyltransferase

^3^Plasmid pYG213 is a deletion derivative of pYG122. Plasmid pYG121, 122, 213 have the ampicillin-resistance gene

^4^Plasmid pYG219 is a derivative of pBR322 having 1.35 kb DNA containing the *oat* gene from pYG213. It has the tetracycline-resistance gene

^5^Plasmid pYG216 is a derivative of pBR322 having 6.85 kb DNA containing the c*nr* gene encoding the classical nitroreductase from strain TA1538. Plasmid pYG216 has the tetracycline-resistance gene

^6^Plasmid pYG233 is a derivative of pBR322 having the *oat* and c*nr* genes. Plasmid pYG233 has the tetracycline-resistance gene

### Review

#### Development of *Salmonella typhimurium* YG strains

In the 1970s, Dr. Bruce N. Ames, University of California, developed a bacterial mutagenicity test (Ames test) and reported that a high percentage of bacterial mutagens in the Ames test are rodent carcinogens [27, 28]. The test is simple, rapid and economical; therefore, large number of environmental chemicals were tested for potential mutagenicity in the Ames tester strains. Typical tester strains of the Ames test are *Salmonella typhimurium* TA98 and TA100, which detect frameshift-type mutagens and base-substitution-type mutagens, respectively [19, 20]. In the same era, Dr. Takashi Sugimura, National Cancer Center in Japan, was interested in the possibility that smoke from broiled fish might be mutagenic and carcinogenic. Dr. Sugimura and his collaborators examined this possibility using the Ames test and isolated many heterocyclic amines as mutagens from pyrolysates of amino acids, proteins, meat or fish as mutagens [6, 29]. Similarly, Dr. Daisuke Yoshida, the Japan Tobacco & Salt Public Cooperation, isolated AαC and MeAαC from pyrolysis products of soybean globulin and Dr. James S. Felton, Lawrence Livermore National Laboratory, U.S.A., identified PhIP and the related chemicals from fried ground beef [9, 14].

Heterocyclic amines require metabolic activation for mutagenesis and carcinogenesis. In general, they are first oxidized by CYP1A2 to *N*-hydroxy derivatives, which are further activated by *O*-acetyltransferase or sulfotransferase to the nitrenium ions, thereby inducing DNA adducts and mutations [30–33]. In the Ames test, these metabolic enzymes are provided as 9,000 x g supernatant of rat liver homogenates (S9) [34]. It must be pointed out, however, *Salmonella typhimurium* used in the Ames test has enzymes involved in metabolic activation. In fact, strain TA98/1,8-DNP_6_ is significantly resistant to the mutagenicity and killing effects of aromatic amines and nitro aromatics, because this strain is devoid of acetyltransferase activity [21].

**Fig. 1:**
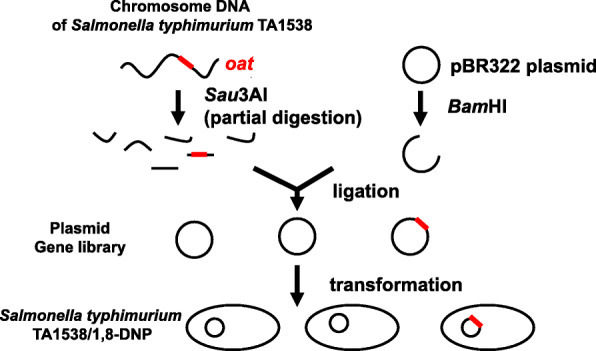
Gene cloning of the *oat* gene encoding *O*-acetyltransferase in *Salmonella typhimurium*. The chromosome DNA of *Salmonella typhimurium* TA1538 was partially digested with Sau3A1 and ligated to BamH1-digested plasmid pBR322, thereby generating a plasmid library of TA1538. Then, the library DNA was introduced into *Salmonella typhimurium* TA1538/1,8-DNP and screened the colonies that could grow on plates without 2-NF but could not grow on plates with 2-NF

In the mid-1980s, we were interested in the metabolic activation mechanisms of chemical carcinogens and cloned the *oat* gene encoding bacterial *O*-acetyltransferase [16, 22]. For this purpose, we constructed a gene library of *Salmonella typhimurium* strain TA1538 with a multicopy-number plasmid pBR322 and introduced the gene library into strain TA1538/1,8-DNP, which is the same as TA98/1,8-DNP_6_ but lacks plasmid pKM101 (Fig. 1). We searched for colonies that could grow on plates without 2-nitrofluorene (2-NF) but could not grow on plates with 2-NF. The principle was that if a plasmid carrying the *oat* gene was introduced into the host strain TA1538/1,8-DNP, the transformants would not grow on plates with 2-NF but grow on plates without 2-NF because 2-NF requires activities of *O*-acetyltransferase for cytotoxicity and mutagenicity. Fortunately, we successfully isolated candidate colonies and confirmed that the sensitivity was maintained after the plasmids extracted from the candidate colonies were introduced to the fresh background of TA1538/1,8-DNP. Plasmid pYG121 and pYG122 were the first isolated plasmids that carried the *oat* gene (Table 1). We then constructed plasmid pYG213, a deletion derivative of pYG122, which contains a 1.35kb-DNA fragment of pYG122 including the *oat* gene. However, pYG213 has the ampicillin-resistance-gene and is incompatible with strains TA98 and TA100, both of which possess pKM101 that confers ampicillin resistance. Therefore, we subcloned the 1.35-kb DNA fragment into the ScaI site of pBR322 and generated pYG219. Subcloning into this site disrupted the ampicillin-resistance gene and permitted the selection of pYG219 in TA98 and TA100. The resulting strains were named as YG1024 and YG1029, respectively [16]. *N*-hydroxy-Glu-P-1 *O*-acetyltransferase activities of TA1538/1,8-DNP harboring pBR322, pYG122, pYG213 or pYG219 were 0, 28.0, 228 or 54.6 nmol/min/mg protein, respectively [16, 23]. Although strain YG1012, which is TA1538/1,8-DNP harboring pYG213, had the highest *O*-acetyltransferase activity, it exhibited lower sensitivity to the mutagenicity of 1-aminonaphthalene + S9, 1-nitropyrene and 1,8-dinitropyrene compared to YG1024 [23]. It suggests that these chemicals require the presence of pKM101 for maximum frameshift mutagenesis. Plasmid pKM101 carries the *mucAB* genes encoding DNA polymerase RI, an error-prone DNA polymerase involved in translesion DNA synthesis [35]. Owing to the possession of pKM101 and the wider range of sensitivity, strain YG1024 is more widely used for mutation assays than strain YG1012 [36]. However, YG1024 showed comparable or slightly lower sensitivity to 2-hydroxy-acetylaminofluorene, Glu-P-1 + S9 and 2-aminoanthracene +S9 compared to YG1012 [23]. It appears that these chemicals are not strongly dependent on the presence of pKM101 for maximum mutagenesis. Later, we constructed plasmid pYG233 carrying the *oat* gene and the *cnr* gene encoding classical nitroreductase [24] and introduced it to strains TA98 and TA100. The resulting strains YG1041 and YG1042, respectively, overexpressed both acetyltransferase and nitroreductase [26]. They were more sensitive to the mutagenicity of nitroaromatics such as 2-NF, 2,6-dinitrotoluene and 1-nitropyrene than YG1024 or YG1029. A possible problem with YG1041 and YG1042 is the extreme sensitivity to the killing effects of nitro, amino and hydroxyamino compounds. The number of revertants increased very sharply and decreased quickly with increasing doses. In addition, the number of spontaneous revertants per plate of YG1041 and YG1042 was higher than that of spontaneous revertants per plate of YG1024 and YG1029, respectively. The high number of spontaneous revertants obscures the weak mutagenicity of chemicals. Therefore, we recommend using these strains along with other strains such as YG1024 and YG1029 to avoid overlooking the mutagenic responses of test chemicals.

### Mutagenicity of heterocyclic amines in YG strains

#### Novel heterocyclic amines

Heterocyclic amines were initially isolated from the pyrolysates of food or food components. Later, they were isolated from various environmental sources such as river water [37], automobile exhaust particles [38], cigarette smoke [39], human excretion [40] and rainwater [41]. Appropriate devices and methods are required to efficiently collect environmental mutagens. In the case of river water, it is critical to effectively collect and concentrate the target molecules from a large volume of water samples because pollutants are present in only minute concentrations. Sakamoto and Hayatsu developed an effective method, i.e., the blue rayon hanging technique, in which blue rayon covalently bound to the blue pigment copper phthalocyanine is hung in the river to specifically adsorb polycyclic planar compounds including heterocyclic amines [42]. The blue rayon absorbing water pollutants, instead of a large volume of water samples, is transferred to laboratories for chemical analyses and mutagenicity assays. Kataoka et al. [43] isolated and identified IQ, Trp-P-1 and AαC in the Danube River in Vienna by the method. The river water samples exhibited higher mutagenicity in YG1024 than in TA98 in the presence of S9 activation, which suggested a significant contribution of the heterocyclic amines to the whole mutagenicity of the water samples (Table 2). The source of the heterocyclic amines in the Danube River may be the emission and discharge from food processing, e.g., smoke sausage, and wood burning. The collection of mutagens in river water by the blue-rayon hanging technique and the subsequent mutagenicity assays with YG1024 were conducted in samples from the Chao Phraya River in Bangkok, Thailand, and the Sumida and Ara Rivers in Tokyo [44]. Similar methods were employed for samples from rivers in Boston, New York, Washington D.C. and Montreal in North America [45]. In the latter case, YG1041 and YG1024 were much more sensitive than TA98 in the presence of S9 plus an NADPH-generating system (S9 mix).

**Table 2:** Mutagenicity of heterocyclic amines in *Salmonella typhimurium* YG strains

Chemical	Strain	Metabolic activation	Remarks	Reference
2-amino-1-methyl-6-phenylimidazo [4,5-*b*]pyridine (PhIP)	TA98, TA98NR, YG1024, TA98/1,8-DNP_6_	W	The sensitivity (induced revertants/nmol) was in the order of YG1024 > TA98 = TA98/1,8-DNP_6_ = TA98NR	[46]
2-amino-1-methyl-6-phenylimidazo [4,5-*b*]pyridine (PhIP)	TA98, YG1024	W	The sensitivity was about 3 times higher in YG1024 than in TA98. The mutagenic potency (induced revertants/μg) was more than 100 times lower than that of MeIQx.	[47]
2-amino-1-methyl-6-phenylimidazo [4,5-*b*]pyridine (PhIP)	YG1024	W	The mutagenicity of PhIP was enhanced up to six times by the presence of ethylparathion, methylparathion or methyl paraoxon.	[48]
2-amino-1-methyl-6-phenylimidazo [4,5-*b*]pyridine (PhIP)	TA98, YG1024	W*	PhIP was negative in both strains in the presence of colon S9 prepared from 3-MC-treated rats.	[49]
2-amino-1-methyl-6-phenylimidazo [4,5-*b*]pyridine (PhIP)	TA1538, YG1019	W	The mutagenic potency (revertants/ng) was in the order of MeIQx = IQ > 4,8-diMeIQx>> > PhIP>MeAαC>AαC in YG1019.	[50]
2-amino-1-methyl-6-phenylimidazo [4,5-*b*]pyridine (PhIP)	YG1024	W*	The mutagenic potency was IQ = MeIQ>Trp-P-1 = MeIQx>PhIP in the presence of HepG2 cell homogenates. The order was unchanged when rat S9 was used although the mutagenic potency was much more enhanced with rat S9.	[51]
2-nitro-1-methyl-6-phenylimidazo [4,5-*b*]pyridine (NO_2_-PhIP)	TA98, TA98NR, YG1024, TA98/1,8-DNP_6_	W/O	The sensitivity (induced revertants/nmol) was in the order of YG1024 = TA98 > TA98/1,8-DNP_6_ > TA98NR.	[46]
2-azido-1-methyl-6-phenylimidazo [4,5-*b*]pyridine (azido-PhIP)	TA98, TA98NR, YG1024, TA98/1,8-DNP_6_	W*	Near UV light was used for activation. The sensitivity (induced revertants/nmol) was in the order of YG1024 = TA98 = TA98NR > TA98/1,8-DNP_6_.	[46]
2-amino-6-methyldipyrido[1,2-*a:*3′,2′-*d*]imidazole (Glu-P-1)	YG1020, YG1024, YG1025, YG1029	W	The sensitivity (induced revertants/nmol) was in the order of YG1024> > YG1020 > YG1029 > YG1025.	[16]
2-amino-6-methyldipyrido[1,2-*a:*3′,2′-*d*]imidazole (Glu-P-1)	TA98, TA98NR, TA98/1,8-DNP_6_, YG1021, YG1024	W	The sensitivity (induced revertants/nmol) was in the order of YG1024 > YG1021 > TA98 > TA98NR > TA98/1,8-DNP_6_.	[36]
2-amino-6-methyldipyrido[1,2-*a:*3′,2′-*d*]imidazole (Glu-P-1)	YG1020, YG1024, YG1012, YG1019	W	The sensitivity (induced revertants/nmol) was in the order of YG1012 > YG1019 = YG1024 > YG1020.	[23]
2-amino-6-methyldipyrido[1,2-*a:*3′,2′-*d*]imidazole (Glu-P-1)	TA98, YG1021, YG1024, YG1041	W	The sensitivity (induced revertants/nmol) was in the order of YG1024 = YG1041 > TA98 > YG1021.	[26]
2-amino-6-methyldipyrido[1,2-*a:*3′,2′-*d*]imidazole (Glu-P-1)	YG1024	W	YG1024 may lose plasmid pYG219 under highly toxic conditions.	[52]
2-amino-6-methyldipyrido[1,2-*a:*3′,2′-*d*]imidazole (Glu-P-1)	YG1006, TA98	W*	Ram seminal vesicle microsomes (prostaglandin H synthase) activated Glu-P-1 for mutagenesis in YG1006.	[53]
2-hydroxyamino-6-methyldipyrido[1,2-*a*:3′,2′-*d*]imidazole (N-OH-Glu-P-1)	YG1020, YG1024, YG1025, YG1029	W/O	The sensitivity (induced revertants/nmol) was in the order of YG1024> > YG1020 > YG1029 > YG1025. S9 was not needed for the mutagenicity.	[16]
2-hydroxyamino-6-methyldipyrido[1,2-*a*:3′,2′-*d]*imidazole (N-OH-Glu-P-1)	TA98, TA98NR, TA98/1,8-DNP_6_, YG1021, YG1024	W/O	The sensitivity (induced revertants/nmol) was in the order of YG1024 > TA98 = YG1021 = TA98NR > TA98/1,8-DNP_6_. S9 was not needed for the mutagenicity.	[36]
2-hydroxyamino-6-methyldipyrido[1,2-*a*:3′,2′-*d*]imidazole (N-OH-Glu-P-1)	TA98, YG1021, YG1024, YG1041	W/O	The sensitivity (induced revertants/nmol) was in the order of YG1041 > YG1024 > YG1021 = TA98.	[26]
2-nitro-6-methyldipyrido[1,2-*a*:3′,2′-*d*]imidazole (NO_2_-Glu-P-1)	TA98, YG1021, YG1024, YG1041	W/O	The sensitivity (induced revertants/nmol) was in the order of YG1041> > YG1024 > YG1021 > TA98.	[26]
2-nitro-6-methyldipyrido[1,2-*a*:3′,2′-*d*]imidazole (NO_2_-Glu-P-1)	TA98, TA98NR, TA98/1,8-DNP_6_, YG1021, YG1024	W/O	The sensitivity (induced revertants/nmol) was in the order of YG1024 > YG1021 > TA98 > TA98/1,8-DNP_6_ > TA98NR.	[36]
3-amino-1,4-dimethyl-5*H*-pyrido[4,3-*b*]indole (Trp-P-1)	YG1024	W*	The mutagenic potency was IQ = MeIQ>Trp-P-1 = MeIQx>PhIP in the presence ofI HepG2 cell homogenates. The order was unchanged when rat S9 was used although the mutagenic potency was much more enhanced with rat S9.	[51]
3-amino-1,4-dimethyl-5*H*-pyrido[4,3-*b*]indole (Trp-P-1)	TA98, YG1024	W	Trp-P-1 was identified in samples from the Danube River in Vienna.	[43]
3-amino-1-methyl-5*H*-pyrido[4,3-*b*]indole (Trp-P-2)	TA98, TA98NR, TA98/1,8-DNP_6_, YG1021, YG1024	W	The sensitivity (induced revertants/nmol) was in the order of TA98/1,8-DNP_6_ > YG1021 = TA98 > YG1024 = TA98NR.	[36]
3-amino-1-methyl-5*H*-pyrido[4,3-*b*]indole (Trp-P-2)	TA98, YG1021, YG1024, YG1041	W	The sensitivity (induced revertants/nmol) was not substantially different among the four strains.	[26]
3-amino-1-methyl-5*H*-pyrido[4,3-*b*]indole (Trp-P-2)	TA98, YG1024	W*	Untreated rat liver S12 fraction was used for metabolic activation. The sensitivity was similar between TA98 and YG1024.	[54]
2-amino-3-methylimidazo[4,5-*f*]quinoline (IQ)	YG1012	W*	Human or rat cytochrome P-450 1A2 plus hydrogen peroxide supported metabolic activation of IQ	[55]
2-amino-3-methylimidazo[4,5-*f*]quinoline (IQ)	TA1538, YG1019	W	The mutagenic potency (revertants/ng) was in the order of MeIQx = IQ > 4,8-diMeIQx>> > PhIP>MeAαC>AαC in YG1019.	[50]
2-amino-3-methylimidazo[4,5-*f*]quinoline (IQ)	TA98, YG1024, TA98/1,8-DNP_6_	W	The sensitivity (induced revertants/nmol) was in the order of YG1024> > TA98> > TA98/1,8-DNP_6_.	[46]
2-amino-3-methylimidazo[4,5-*f*]quinoline (IQ)	YG1024	W*	Ram seminal vesicle microsomes(supplemented with arachidonic acid) activated IQ for mutagenesis.	[56]
2-amino-3-methylimidazo[4,5-*f*]quinoline (IQ)	YG1024, TA98	W	The sensitivity (induced revertants/nmol) was in the order of YG1024 > TA98.	[57]
2-amino-3-methylimidazo[4,5-*f*]quinoline (IQ)	YG1024	W*	The mutagenic potency was IQ = MeIQ>Trp-P-1 = MeIQx>PhIP in the presence of HepG2 cell homogenates. The order was unchanged when rat S9 was used although the mutagenic potency was much more enhanced with rat S9.	[51]
2-amino-3-methylimidazo[4,5-*f*]quinoline (IQ)	YG1024	W*	The C8-dG-IQ-adduct *N*-(deoxyguanosin-8-yl)-IQ was the major adduct when IQ was incubated with YG1024 either in ovine seminal vesicle cells (prostaglandin H synthase) or hepatocytes (monooxygenases).	[58]
2-amino-3-methylimidazo[4,5-*f*]quinoline (IQ)	YG1024	W	Urinary metabolites of IQ-treated rats were investigated with improved extraction methods and assay conditions.	[59]
2-amino-3-methylimidazo[4,5-*f*]quinoline (IQ)	YG1006, TA98	W*	Ram seminal vesicle microsomes (prostaglandin H synthase) activated IQ for mutagenesis in YG1006.	[53]
2-amino-3-methylimidazo[4,5-*f*]quinoline (IQ)	YG1024	W	The mutagenicity of IQ was enhanced about two times by the presence of methyl parathion and methyl paraoxon.	[48]
2-amino-3-methylimidazo[4,5-*f*]quinoline (IQ)	YG1012	W*	Ram seminal vesicle microsomes (prostaglandin H synthase) activated IQ for mutagenesis in YG1012.	[25]
2-amino-3-methylimidazo[4,5-*f*]quinoline (IQ)	TA98, YG1024	W	IQ was identified in samples from the Danube River in Vienna.	[43]
2-amino-3-methylimidazo[4,5-*f*]quinoline (IQ)	YG1024, TA98	W	The mutagenic potency of IQ (revertants/μg) was more than 5 times higher than that of IQx.	[60]
2-nitro-3-methylimidazo[4,5-*f*]quinoline (NO_2_-IQ)	TA98, YG1024, TA98/1,8-DNP_6_	W/O	The sensitivity (induced revertants/nmol) was in the order of YG1024> > TA98> > TA98/1,8-DNP_6_.	[46]
2-nitro-3-methylimidazo[4,5-*f*]quinoline (NO_2_-IQ)	YG1024	W/O	NO_2_-IQ and NO-IQ exhibited similar mutagenicity to YG1024.	[56]
2-nitro-3-methylimidazo[4,5-*f*]quinoline (NO_2_-IQ)	YG1012, YG1024, YG1024NR	W/O	NO_2_-IQ was a metabolite of IQ by ram seminal vesicle microsomes (prostaglandin H synthase). YG1012 exhibited similar or slightly higher sensitivity to NO_2_-IQ than YG1024.	[25]
2-nitroso-3-methylimidazo[4,5-*f*]quinoline (NO-IQ)	YG1024	W/O	NO_2_-IQ and NO-IQ showed similar mutagenicity to YG1024.	[56]
7-hydroxy-2-amino-3-methylimidazo[4,5-*f*]quinoline (7-OH-IQ)	YG1012, YG1024NR	W/O	7-OH-IQ was a possible metabolite of IQ by ram seminal vesicle microsomes. The mutagenicity was substantially lower than that of NO_2_-IQ.	[25]
2, 2′-azo-bis-3-methylimidazo[4,5-*f*]quinoline (azo-IQ)	YG1024, TA98	W/O	azo-IQ was a metabolite of IQ in the presence of ram seminal vesicle microsomes. The mutagenicity was much weaker than NO-IQ or NO_2_-IQ.	[56]
2-amino-3-methylimidazo[4,5-*f*]quinoxaline (IQx)	YG1024, TA98	W	The mutagenic potency of IQx (revertants/μg) was more than 50 times higher than that of 1-methynaphtho[2,3-*d*]imidazole-2-amine (Linear-NI).	[60]
2-amino-3,4-dimethylimidazo[4,5-*f*]quinoline (MeIQ)	YG1024, TA98	W	YG1024 may lose plasmid pYG219 under highly toxic conditions.	[52]
2-amino-3,4-dimethylimidazo[4,5-*f*]quinoline (MeIQ)	YG1024, TA98	W	The sensitivity (induced revertants/nmol) was in the order of YG1024 > TA98.	[57]
2-amino-3,4-dimethylimidazo[4,5-*f*]quinoline (MeIQ)	YG1024, TA98	W*	Untreated rat liver S12 fraction was used for metabolic activation. The sensitivity was similar between TA98 and YG1024 because of the high toxicity.	[54]
2-amino-3,4-dimethylimidazo[4,5-*f*]quinoline (MeIQ)	YG1024	W*	The mutagenic potency was IQ = MeIQ>Trp-P-1 = MeIQx>PhIP in the presence of HepG2 cell homogenates. The order was unchanged when rat S9 was used although the mutagenic potency was much more enhanced with rat S9.	[51]
2-amino-3,4-dimethylimidazo[4,5-*f*]quinoline (MeIQ)	YG1006, TA98	W*	Ram seminal vesicle microsomes (prostaglandin H synthase) activated MeIQ for mutagenesis in YG1006.	[53]
2-amino-3,8-dimethylimidazo[4,5-*f*]quinoxaline (MeIQx)	TA1538, YG1019	W	The mutagenic potency (revertants/ng) was in the order of MeIQx = IQ > 4,8-diMeIQx>> > PhIP>MeAαC>AαC in YG1019.	[50]
2-amino-3,8-dimethylimidazo[4,5-*f*]quinoxaline (MeIQx)	YG1024, TA98	W*	MeIQx was mutagenic to YG1024 in the presence of human liver microsomes. YG1024 was more sensitive than TA98. N-OH-MeIQx was a major oxidation product by human liver microsomes.	[62]
2-amino-3,8-dimethylimidazo[4,5-*f*]quinoxaline (MeIQx)	YG1024, TA98	W	The sensitivity (induced revertants/nmol) was in the order of YG1024 > TA98.	[57]
2-amino-3,8-dimethylimidazo[4,5-*f*]quinoxaline (MeIQx)	YG1024	W*	The mutagenic potency was IQ = MeIQ>Trp-P-1 = MeIQx>PhIP in the presence of HepG2 cell homogenates. The order was unchanged when rat S9 was used although the mutagenic potency was much more enhanced with rat S9.	[51]
2-amino-3,8-dimethylimidazo[4,5-*f*]quinoxaline (MeIQx)	YG1024, TA98	W	The sensitivity (induced revertants/μg) was about 12 times higher in YG1024 than in TA98.	[47]
2-amino-3,8-dimethylimidazo[4,5-*f*]quinoxaline (MeIQx)	YG1024	W	The mutagenicity of MeIQx was suppressed by the urinary phenolics in humans.	[61]
2-hydroxyamino-3,8-dimethylimidazo[4,5-*f*]quinoxaline (*N*-OHMeIQx)	TA98, YG1024, TA98/1,8-DNP_6_	W/O	The sensitivity (induced revertants/nmol) was in the order of YG1024 > TA98 > TA98/1,8-DNP_6_. N-OH-MeIQx was identified as a major metabolite of MeIQx by human CYP1A2.	[62]
2-amino-4-hydroxymethyl-3,8-dimethylimidazo[4,5-*f*]quinoxaline (4-CH_2_OH-8-MeIQx)	YG1024, TA98, TA100	W	The sensitivity was in the order of YG1024 > TA98> > TA100.	[63]
2-amino-3,4,8-trimethylimidazo[4,5-*f*]quinoxaline (4,8-DiMeIQx)	TA1538, YG1019	W	The mutagenic potency (revertants/ng) was in the order of MeIQx = IQ > 4,8-diMeIQx>> > PhIP>MeAαC>AαC in YG1019.	[50]
2-amino-1,7,9-trimethylimidazo[4,5-*g*]quinoxaline (7,9-diMeIQx)	YG1024, TA98	W	The sensitivity was in the order of YG1024 > TA98. The mutagenic potency (induced revertants/μg) of 7,9-diMeIQx was 250 times lower than those of MeIQx and 4,8-diMeIQx and 3 times lower than that of PhIP.	[64]
2-amino-1,6-dimethylimidazo[4,5-*g*]quinoxaline (6-MeIQx)	YG1024, TA98	W	The sensitivity was about 8 fold higher in YG1024 than in TA98. 6-MeIQx was a weak mutagen.	[47]
2-amino-1,7-dimethylimidazo[4,5-*g*]quinoxaline (7-MeIQx)	YG1024, TA98	W	The sensitivity was about 16 times higher in YG1024 than in TA98. The mutagenic potency (induced revertants/μg) was more than 4000 times lower than that of MeIQx.	[47]
2-amino-1,7,9-trimethylimidazo[4,5-*g*]quinoxaline (7,9-diMeIQx)	YG1024, TA98	W	The sensitivity was about 4 times higher in YG1024 than in TA98. The mutagenic potency (induced revertants/μg) was more than 100 times lower than that of MeIQx.	[47]
9-(4′-aminophenyl)-9*H*-pyrido[3,4-*b*]indole (aminophenylnorharman, APNH)	TA98, YG1024, TA100, YG1029	W	APNH was formed from aniline and norharman in the presence of S9 mix. The sensitivity was YG1024> > TA98> > YG1029 > TA100. The mutagenic potency of APNH (revertants/μg) was comparable to those of MeIQx and Glu-P-1.	[65]
9-(4′-hydroxyaminophenyl)-9*H*-pyrido[3,4-*b*]indole (hydroxyaminophenylnorharman)	YG1024	W/O	*N-*hydroxy derivative of APNH.	[65]
9-(4′-amino-3′-methylphenyl)-9*H*-pyrido[3,4-*b*]indole (amino-3′-methylphenylnorharman)	TA98, TA100, YG1024, YG1029	W	Amino-3′-methylphenylnorharman was formed from aniline and *o*-toluidine in the presence of S9 mix.. The sensitivity was YG1024> > TA98 > YG1029 > TA100. The mutagenic potency (revertants/μg) was weaker than that of aminophenylnorharman.	[66]
9-(4′-amino-2′-methylphenyl)-9*H*-pyrido[3,4-*b*]indole (amino-2′-methylphenylnorharman)	TA98, TA100, YG1024, YG1029	W	Amino-2′-methylphenylnorharman was formed from aniline and *m*-toluidine in the presence of S9 mix. The sensitivity was YG1024> > TA98 > YG1029 > TA100. The mutagenic potency (revertants/μg) was weaker than that of amino-3′-methylphenylnorharman.	[66]
5-amino-6-hydroxy-8*H*-benzo[6,7]azepino[5,4,3-*de*]quinolin-7-one (ABAQ)	TA98, TA100, YG1024, YG1029	W	ABAQ was formed by the Maillard reaction of glucose and amino acids. The sensitivity was YG1024> > TA98 > YG1029 > TA100. The mutagenic potency of ABAQ (revertants/μg) was comparable to that of PhIP.	[67]
2-amino-9*H*-pyrido[2,3-*b*]indole (AαC)	TA1538, YG1019	W	The mutagenic potency (revertants/ng) was in the order of MeIQx = IQ > 4,8-diMeIQx>> > PhIP>MeAαC>AαC in YG1019.	[50]
2-amino-9*H*-pyrido[2,3-*b*]indole (AαC)	YG1019, TA1538	W	AαC is a mutagen detected in panfried or grilled meat. YG1019 exibited higher sensitivity to AαC than TA1538.	[68]
2-amino-9*H*-pyrido[2,3-*b*]indole (AαC)	TA98, YG1024	W	AαC was identified in samples from the Danube River in Vienna.	[43]
2-nitro-9*H*-pyrido[2,3-*b*]indole (NαC)	YG1019	W/O	NαC was a direct-acting mutagen. The mutagenic potency in the absence of S9 was lower than that of AαC in the presence of S9.	[68]
2-amino-3-methyl-9*H*-pyrido[2,3-*b*]indole (MeAαC)	TA1538, YG1019	W	The mutagenic potency (revertants/ng) was in the order of MeIQx = IQ > 4,8-diMeIQx>> > PhIP>MeAαC>AαC in YG1019.	[50]
1-methylimidazo[4,5-*b*][1,8]naphthyridin-2-amine (compound 1)	YG1024, TA98	W	The mutagenic potency (revertants/μg) was IQ> > IQx> > Linear-NI > compound 2 > compound 5 > compound 3 = compound 4 > compound 1. The sensitivity was YG1024 > > TA98.	[60]
1-methylimidazo[4,5-*b*][1,7]naphthyridin-2-amine (compound 2)	YG1024, TA98	W		
1-methylimidazo[4,5-*b*][1,6]naphthyridin-2-amine (compound 3)	YG1024, TA98	W		
1-methylimidazo[4,5-g][1,5]naphthyridin-2-amine (compound 4)	YG1024, TA98	W		
1-methylimidazo[4,5-*b*]quinoline-2-amine (compound 5)	YG1024, TA98	W		
1-methynaphtho[2,3-*d*]imidazole-2-amine (linear-NI)	YG1024, TA98	W		
2-[2-(acetylamino)-4-[bis(2-methoxyethyl)amino]-5-methoxyphenyl]-5-amino-7-bromo-4-chloro-2*H*-benzotriazole (PBTA-1)	YG1024	W	PBTA-1 was a novel aromatic amine mutagen isolated from river water in Kyoto. The mutagenicity potency (revertants/μg) was comparable to that of Glu-P-1.	[69]
2-[2-(acetylamino)-4-[bis(2-methoxyethyl)amino]-5-methoxyphenyl]-5-amino-7-bromo-4-chloro-2*H*-benzotriazole (PBTA-1)	TA98, TA100, YG1024, YG1029	W	The sensitivity was in the order of YG1024 > > TA98 > YG1029> > TA100.	[70]
2-[(2-bromo-4,6-dinitrophenyl)azo]-4-methoxy-5-[bis(2-methoxyethyl)amino]acetoanilide (AZO DYE-1)	TA98, TA100, YG1024, YG1029	W	AZO DYE-1 was converted to PBTA-1 through deClPBTA-1. The potency of AZO DYE-1 (revertants/μg) was 1000-fold lower than that of PBTA-1.	[70]
2-[2-(acetylamino)-4-[bis(2-methoxyethyl)amino]-5-methoxyphenyl]-6-amino-4-bromo-2*H*-benzotriazole (deClPBTA-1)	TA98, TA100, YG1024, YG1029	W	deClPBTA-1 was an intermediate from AZO-DYE-1 to PBTA-1. The potency of deClPBTA-1 (revertants/μg) was lower than that of PBTA-1 but higher than that of AZO-DYE-1.	[70]
2-[2-(acetylamino)-4-[*N*-(2-cyanoethyl)ethylamino]-5-methoxyphenyl]-5-amino-7-bromo-4-chloro-2*H*-benzotriazole (PBTA-2)	TA98, TA100, YG1024, YG1029	W	PBTA-2 was a novel aromatic amine mutagen isolated from river water in Kyoto. The sensitivity was YG1024> > TA98. The mutagen may be produced from an azo dye in dyeing factories and treatment at sewage plants.	[71]
2-[(2-bromo-4,6-dinitrophenyl)azo]-5-[*N*-(2-cyanoethyl)ethylamino]-4-methoxyacetoanilide (AZO DYE-2)	TA98, TA100, YG1024, YG1029	W	AZO DYE-2 was converted to PBTA-2 through deClPBTA-2. The potency of AZO DYE-2 (revertants/μg) was 1000-fold lower than that of PBTA-1.	[71]
2-[2-(acetylamino)-4-[*N*-(2-cyanoethyl)ethylamino]-5-methoxyphenyl]-6-amino-4-bromo-2*H*-benzotriazole (deClPBTA-2)	TA98, TA100, YG1024, YG1029	W	deClPBTA-2 was an intermediate from AZO-DYE-2 to PBTA-2. The potency of deClPBTA-2 (revertants/μg) was lower than PBTA-2 but higher than AZO-DYE-2.	[71]
PBTA-1, PBTA-2	YG1024	W	PBTA-1 and PBTA-2 were released from sewage plants into the Yodo river in Japan.	[72]
2-[2-(acetylamino)-4-[(2-hydroxyethyl)amino]-5-methoxyphenyl]-5-amino-7-bromo-4-chloro-2*H*-benzotriazole (PBTA-3)	TA98, YG1024	W	The sensitivity was YG1024> > TA98. The mutagenic potency of PBTA-3(revertants/μg) was comparable to those of PBTA-1 and PBTA-2.	[73]
2-[2-(acetylamino)-4-amino-5-methoxyphenyl]-5-amino-7-bromo-4-chloro-2*H*-benzotriazole (PBTA-4)	TA98, TA100, YG1024, YG1029	W	The sensitivity was YG1024> > TA98> > YG1029 > TA100. The mutagenic potency of PBTA-4 (revertants/μg) was about twice as high as those of PBTA-1, PBTA-2 and PBTA-3.	[74]
2-[2-(acetylamino)-4-amino-5-methoxyphenyl]-6-amino-4-bromo-2*H*-benzotriazole (non-ClPBTA-4)	TA98, TA100, YG1024, YG1029	W	The sensitivity was YG1024> > YG1029 > TA98> > TA100. The mutagenic potency (revertants/μg) was about 20 times lower than that of PBTA-4.	[74]
5-amino-2-[(2-bromo-4,6-dinitrophenyl)azo]-5-amino-4-methoxyacetoanilide (AZO DYE-4)	TA98, TA100, YG1024, YG1029	W	AZO DYE-4 was converted to PBTA-4 through deClPBTA-4. The potency of AZO DYE-4 (revertants/μg) was more than 2000-fold lower than that of PBTA-4.	[74]
2-[4-[bis(2-acetoxyethyl)amino]-2-(acetylamino)-5-methoxyphenyl]-5-amino-7-bromo-4-chloro-2*H*-benzotriazole (PBTA-5)	TA98, TA100, YG1024, YG1029	W	The sensitivity was YG1024> > TA98> > YG1029 > TA100. The mutagenic potency of PBTA-5 (revertants/μg) was about 10 times lower than that of PBTA4.	[75]
2-[2-(acetylamino)-4-[bis-(2-hydroxyethyl)amino]-5-methoxyphenyl]-5-amino-7-bromo-4-chloro-2*H*-benzotriazole (PBTA-6)	TA98, TA100, YG1024, YG1029	W	The sensitivity was YG1024> > TA98> > YG1029 > TA100. The mutagenic potency of PBTA-6 (revertants/μg) was two to three times lower than that of PBTA5.	[75]
PBTA-3, PBTA-4, PBTA-6	YG1024, YG1029	W	PBTA-3, PBTA-4 and PBTA-6 substantially contributed to the mutagenicity of river water in Fukui, Japan.	[76]
PBTA-2, PBTA-3, PBTA-4, PBTA-6	YG1024	W	PBTA2, PBTA-3, PBTA-4 and PBTA-6 were generated in a sawage treatment plant and released to the Uji River, Japan.	[77]
2-[2-(acetylamino)-4-(diethylamino)-5-methoxyphenyl]-5-amino-7-bromo-4-chloro-2*H*-benzotriazole (PBTA-7)	TA98, TA100, YG1024, YG1029	W	PBTA-7 was detected in river water In Japan. The sensitivity was YG1024> > TA98> > YG1029 > TA100. The mutagenic potency (revertants/nmol) was comparable to that of PBTA-1.	[78]
2-[2-(acetylamino)-4-(diallylamino)-5-methoxyphenyl]-5-amino-7-bromo-4-chloro-2*H*-benzotriazole (PBTA-8)	TA98, TA100, YG1024, YG1029	W	PBTA-8 was detected in river water In Japan. The sensitivity was YG1024> > TA98> > YG1029 > TA100. The mutagenic potency (revertants/nmol) was comparable to that of PBTA-1.	[78]
PBTA-1, PBTA-2, PBTA-3, PBTA-4, PBTA-6, PBTA-7, PBTA-8	YG1024	W	About 5 kg PBTA-type mutagens are released per year from sewage plants in the Yodo river in Japan.	[79]
2-[2-(acetylamino)-4-[(2-hydroxyethyl)amino]-5-methoxyphenyl]-6-amino-4-bromo-2*H*-benzotriazole (non-ClPBTA-3)	TA98, TA100, YG1024, YG1029	W	The sensitivity was YG1024> > TA98> > YG1029 > TA100. The mutagenic potency (revertants/μg) was more than 10 times lower than that of PBTA-3.	[80]
2-[2-(acetylamino)-4-(diethylamino)-5-methoxyphenyl]-6-amino-4-bromo-2*H*-benzotriazole (non-ClPBTA-7)	TA98, TA100, YG1024, YG1029	W	The sensitivity was YG1024> > TA98> > YG1029 > TA100. The mutagenic potency (revertants/μg) was more than 5 times lower than that of PBTA-7.	[80]

W: metabolic activation with S9 prepared from inducer-treated rats

W*: metabolic activation with enzyme sources or methods other than S9 prepared from inducer-treated rats

W/O: without metabolic activation

Research on mutagens in river water led to the discovery of a novel class of heterocyclic amines. Nukaya et al. employed the blue rayon hanging technique for the collection of samples at sites below sewage plants of the Nishitakase River in Kyoto, Japan, and identified a novel mutagen, i.e., 2-[2-(acetylamino)-4-[bis(2-methoxyethyl)amino]-5-methoxyphenyl]-5-amino-7-bromo-4-chloro-2*H*-benzotriazole (PBTA-1) [69]. PBTA-1 was highly mutagenic in YG1024 in the presence of S9 mix, and the mutagenic potency (revertants per μg) was equivalent to that of Glu-P-1. For the reason that there are several dye factories in Kyoto, PBTA-1 is probably produced by the treatment of wastewater from dye factories in the sewage plants. In fact, PBTA-1 can be formed from dinitrophenylazo dye used as an industrial material in textile dyeing by reduction and chlorination [70]. PBTA-1 analogs, i.e., PBTA-2, PBTA-3, PBTA-4, PBTA-5, PBTA-6, PBTA-7 and PBTA-8, were later isolated from rivers in Kyoto and Aichi, Japan [71, 73–75, 78]. All these chemicals were mutagenic in YG1024 in the presence of S9 mix and the order of mutagenic potency was PBTA-4 > PBTA-2=PBTA-3 > PBTA-1 > PBTA-5 > PBTA-6 = PBTA-8 > PBTA-7. Despite the potent mutagenicity in the Ames test, the carcinogenicity of PBTAs in rodents has not been reported.

Another novel heterocyclic amine was isolated as a mutagen generated by the Maillard reaction of glucose and amino acids. Nishigaki et al. incubated mixtures of glucose and tryptophan with or without the Fenton reagent and showed that the reaction produced a novel mutagen, i.e., 5-amino-6-hydroxy-8*H*-benzo[6,7]azepino[5,4,3-*de*]quinolin-7-one (ABAQ) [67]. This compound was more mutagenic in YG1024 than in TA98 in the presence of S9 mix and the mutagenic potency was comparable to that of PhIP. ABAQ was mutagenic in the liver of *gpt* delta mice (further detailed provided in the section of “*in vivo* mutagenicity of heterocyclic amines in transgenic rodents”).

#### Metabolic activation of heterocyclic amines

Heterocyclic amines require metabolic activation for mutagenesis via CYP enzymes and either *O*-acetyltransferase or sulfotransferase. As expected, strain YG1024 overexpressing the acetyltransferase exhibited higher sensitivity, i.e., more induced revertants per nmol or μg of chemical, than strain TA98. In fact, YG1024 showed more than 10 times higher sensitivity than TA98 for Glu-P-1, IQ, MeIQ, MeIQx and PBTA-1 [36, 46, 57, 62, 70]. However, YG1024 exhibited similar or only slightly higher sensitivity to PhIP and Trp-P-2 than TA98, suggesting that these heterocyclic amines are not activated by acetyltransferase [46]. Consistent with this, Wu et al. reported that CHO UV-5 cells expressing mouse CYP1A2 and human *N*-acetyltransferase did not exhibit any significant sensitivity or genotoxicity to PhIP [81]. Wu et al. reported in the next paper that CHO UV5 cells expressing mouse CYP1A2 and human aryl sulfotransferases, i.e., HAST1 or HAST3, exhibited higher sensitivity to the killing effects of PhIP than CHO UV5 cells expressing only mouse CYP1A2 [82]. Thus, *N*-hydroxy-PhIP may be activated by sulfotransferase rather than acetyltransferase.

Knasmüller et al. examined the comparative mutagenicity of several heterocyclic amines with strain YG1024 and reported that IQ and MeIQ were the most potent mutagens followed by MeIQx and Trp-P-1 and PhIP was the weakest mutagen [51]. This order was basically the same when strain TA98 was used [6]. Part of the reason for the weak mutagenicity of PhIP in strains YG1024 and TA98 may be its low dependency on acetyltransferase for the metabolic activation.

The crystal structure of Salmonella acetyltransferase was determined at 2.8Å resolution, and it was revealed that a Cys-His-Asp catalytic triad is involved in the catalytic mechanism [83]. The critical Cys residue is conserved between the acetyltransferase of *Salmonella typhimurium* and mammalian acetyltransferases NAT1 and NAT2 [84]. Both acetyltransferases of *Salmonella typhimurium* and mammals catalyze *N*-acetylation (usually inactivation) and *O*-acetylation (usually activation) of heterocyclic amines and the *N*-hydroxy derivatives [85]. Mammalian NAT1 and NAT2 are polymorphic and epidemiological studies suggest that the polymorphisms modify the risk of developing various cancers such as urinary bladder, colorectal and breast cancers.

In addition to CYP enzymes, prostaglandin-H synthase activates several heterocyclic amines. This enzyme is an arachidonic acid-dependent peroxidase and is suggested to be involved in the metabolic activation of xenobiotics in extrahepatic tissues. Ram seminal vesicle microsomes, a rich source of prostaglandin-H synthase, activate IQ and MeIQ for mutagenesis [53, 56]. The mutagenicity was more sensitively detected in YG strains overexpressing Salmonella acetyltransferase, i.e., YG1006 (TA1538/1,8-DNP with pYG121) and YG1024, than in TA98. The primary mutagenic metabolite of IQ by prostaglandin-H synthase is nitro-IQ, while *N*-hydroxy derivatives are the active metabolites of IQ and MeIQx by CYP enzymes [25, 33, 56, 62]. Since nitro-IQ and *N*-hydroxy IQ are further activated by acetyltransferase, the same DNA adduct, i.e., C8-dG-IQ-adduct, is formed in DNA when YG1024 is treated with prostaglandin-H synthase-oxidized IQ or hepatocyte-exposed IQ [58].

#### Co-mutagenic effects

Humans are exposed to not a single chemical but a variety of chemical agents simultaneously. In this regard, modulating effects of chemicals are important for the risk estimation of environmental mutagens. Nagao et al. reported interesting observations that norharman, which is not mutagenic in the Ames test, becomes mutagenic when incubated with non-mutagenic aromatic amines such as aniline, *o*-toluidine or *m*-toluidine in the presence of S9 mix [86]. Later, it was revealed that co-incubation of norharman and aniline with S9 mix produces a novel heterocyclic amine, i.e., 9-(4’-aminophenyl)-9*H*-pyrido[3,4-*b*]indole (aminophenylnorharman, APNH), and the *N*-hydroxy metabolite, i.e., 9-(4’-hydroxyaminophenyl)-9*H*-pyrido[3,4-*b*]indole (hydroxyaminophenylnorharman, N-OH-APNH) [65]. APNH is mutagenic in strains TA98 and YG1024 only when S9 mix is present, while N-OH-APNH is mutagenic without S9 activation. Both chemicals yielded the same DNA adducts in the DNA of YG1024. This strain showed approximately 10 times higher sensitivity to APNH and *N*-OH-APNH than TA98. The mutagenic potency of APNH was comparable to those of MeIQx and Glu-P-1. Similarly, incubation of norharman and *o*-toluidine or *m*-toluidine in the presence of S9 mix generates 9-(4'-amino-3'-methylphenyl)-9*H*-pyrido[3,4-*b*]indole (amino-3'-methylphenylnorharman) and 9-(4'-amino-2'-methylphenyl)-9*H*-pyrido[3,4-*b*]indole (amino-2'-methylphenylnorharman), respectively [66]. These results suggest that non-mutagenic chemicals may become mutagenic when combined.

### Development of *gpt* delta transgenic rodents for mutagenicity assays in vivo

In the late 1980s and the early 1990s, two transgenic mouse models were developed with *E. coli lacZ* or *lacI* as reporter genes for mutations *in vivo* [87, 88]. In these mouse models, i.e., Muta Mouse with *lacZ* and Big Blue Mouse with *lacI*, the λ phage DNAs with the reporter gene were integrated into the chromosome of all the cells of mice [17]. After the mice are treated with chemical agents, the phage is rescued as phage particles from the mouse genome of various organs and tissues by *in vitro* packaging reactions. The rescued phages are introduced into indicator *E. coli* strains to select mutant plaques by color selection, i.e., visual search of colorless plaques in Muta Mouse or blue color plaques in Big Blue Mouse in more than 100,000 background plaques. Transgenic mouse mutagenicity assays allow detection of mutations in any organs or tissues of mice including the liver, lung, bone marrow or testis. However, color selection is time-consuming and expensive because the visual search of plaques of different color is laborious and the chromogenic agent X-gal is expensive. To overcome this limitation, a positive selection with the *cII* gene of phage λ has been developed [89]. The *cII* gene encodes a repressor protein that controls the lysogenic and lytic cycle of λ. Mutations in the *cII* gene can be positively identified with an indicator *E. coli* strain deficient in Hfl protease. In the *hfl*^-^ strain, only λ phage with inactive *cII* can form plaques at 24^o^C. In contrast, all the rescued λ phage can form plaques at 37^o^C regardless of the status of *cII*. Thus, the mutant frequency (MF) can be calculated by dividing the number of plaques formed at 24^o^C by the number of plaques formed at 37^o^C and the dilution factor. The coding size of the *cII* gene is approximately 300 base pairs (bps), which are approximately 1/10 of *lacZ* and 1/3 of *lacI*. Thus, DNA sequencing analysis of the mutants is feasible. The *cII* selection detects point mutations, i.e., base substitutions and frameshifts, but not large deletions. In addition, the *cII* selection is applicable to both Muta Mouse and Big Blue Mouse. Later, Big Blue Rat was developed with the same λ phage DNA, i.e., λ LIZ DNA, with the *lacI* and *cII* genes [90].

**Fig. 2:**
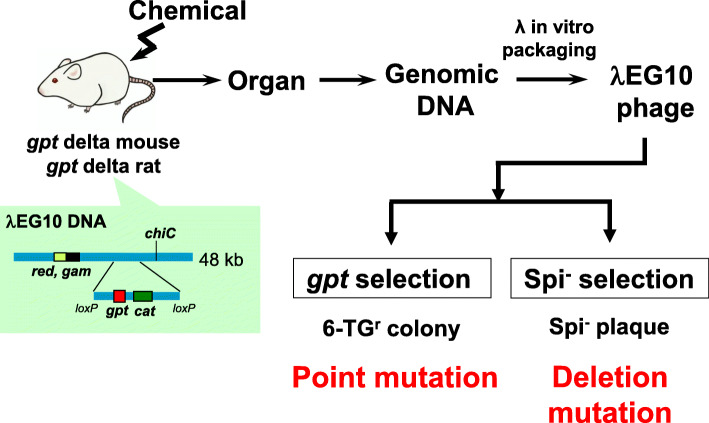
Protocols of *gpt* delta transgenic rodent mutation assays. *Gpt* delta mice or rats are exposed to chemicals by feeding, gavage or others. Then, the genomic DNA is extracted from various organs or tissues to recover λ phage EG10 particles by λ packaging reactions. Then, the rescued phages are introduced to indicator *E. coli* for *gpt* selection and for Spi^−^ selection that detect point mutations and deletion mutations, respectively. DNA is extracted from 6-TG-resistant colonies or Spi^−^ plaques for DNA sequencing

In the mid-1990s, we developed another transgenic mouse model named *gpt* delta by introducing λEG10 DNA into fertilized eggs of C57BL/6J mice [91]. λEG10 DNA was integrated into a single site of the mouse chromosome 17 [92, 93]. A feature of the transgenic mutation assay is the incorporation of two distinct selections for detecting different types of mutations, i.e., *gpt* selection for point mutations and Spi^-^ selection for deletions (Fig. 2) [17]. The *gpt* selection uses the *gpt* gene of *E. coli* as a reporter gene for mutations. The *gpt* gene is a bacterial counterpart of the human *Hprt* gene and encodes guanine phosphoribosyltransferase. When the *gpt* gene is inactivated by mutations, *E. coli* cells can survive on the plates containing 6-thioguanine (6-TG), whereas *E. coli* cells with the wild-type *gpt* gene cannot survive on the plates because they phosphoribosylate 6-TG to a toxic substance, i.e., 6-TGMP. Thus, the *gpt* selection is a positive selection. The coding size of the *gpt* gene is 456 bp, which is convenient for DNA sequencing analysis. The Spi^-^ selection positively detects deletion mutations in λ phage [94]. The selection name Spi^-^ stands for “sensitive to P2 interference”. This selection takes advantage of the restricted growth of the wild-type λ phage in P2 lysogen, which is *E. coli* cells carrying prophage P2 in the chromosome. Only mutant λ deficient in the functions of both the *gam* gene and the *redBA* genes can grow well in P2 lysogens and display the Spi^-^ phenotype. Because the *gam* gene and the *redBA* genes are located side by side in the λ genome, inactivation of both functions is most likely to be induced by deletions in the region. Because of the size limitation of the λ phage in *in vitro* packaging reactions, the size of deletions detectable by the selection is less than 10 kb. However, tandem array of multiple copies of 48-kb λEG10 DNA in the chromosome amounts to a potential target of more than 1 mega bps. Deletion mutations with a molecular size of more than 1 kb were detected by the Spi^-^ selection in various organs such as the liver, spleen, kidney or brain of the mice irradiated with heavy-ions, gamma-rays or X-rays [95–97]. Ultraviolet-B irradiation and treatment with mitomycin C also induced large deletions in the epidermis and bone marrow, respectively [98, 99]. The molecular nature of the deletion mutations can be characterized by DNA sequencing of the mutated *gam* and *redBA* region [100]. Some of the Spi^-^ large deletions have junctions of two broken ends overlapping with short homologous sequences, while others have flush ends. It suggests that non-homologous end-joining plays an essential role in the induction of deletion mutations. The Spi^-^ selection also detects -1 frameshifts in the *gam* gene that interfere with the start of translation of the downstream *redBA* genes [95]. The -1 frameshifts mostly occur in run sequences such as AAAAAA to AAAAA in the *gam* gene, and this type of mutation accounts for most of the spontaneous Spi^-^ mutations.

In the early 2000s, Hayashi et al. introduced λEG10 DNA into fertilized eggs of Sprague-Dawley (SD) rats and established SD *gpt* delta rats [101]. λEG10 DNA was integrated into a single site of the chromosome 4 [93]. The SD *gpt* delta rats were crossed with Fischer 344 (F344) rats for 15 generations and established F344 *gpt* delta rats [102]. Unlike *gpt* delta mice, which have λEG10 DNA in both alleles of chromosome 17, *gpt* delta rats are heterozygous, where λEG10 is integrated into only one allele of chromosome 4. This is because homozygous rats are defective in tooth development and cannot survive after weaning. To overcome this limitation, a new homozygous *gpt* delta rat strain was established in the genetic background of Wistar Hannover [103]. In the new version of *gpt* delta rat, λEG10 was integrated into both alleles of chromosome 1 and exhibited a significantly higher packaging efficiency than the heterozygous *gpt* delta rats. The average of spontaneous *gpt* and Spi^-^ MFs in the liver of heterozygous and new homozygous *gpt* delta rats are 4.4-6.5 x 10^-6^ and 2.8-5.5 x 10^-6^, respectively, which are significantly lower than those of the *lacI* and *cII* genes [104]. The low frequencies of spontaneous MFs of *gpt* and Spi^-^ are similar to those of *gpt* delta mice. Transgenic mouse and rat mutation assays with *gpt* delta mouse/rat, Big Blue mouse/rat and Muta Mouse are recommended for regulatory genotoxicity assays *in vivo* in OECD Test Guideline 488 [105]. For the reason that rats are more frequently used for toxicological studies and cancer bioassays than mice, the transgenic rat mutation assays are expected to be combined with 28-day repeated-dose toxicity studies [106].

### In vivo mutagenicity of heterocyclic amines in transgenic rodents

#### Organ specificity and gender difference

PhIP is the most abundant mutagenic and carcinogenic heterocyclic amine produced in cooked meat and fish [14]. It induces colon and prostate cancers in male F344 rats and mammary cancer, but not colon cancer, in female rats [107, 108]. Okonogi et al. [109] examined the mutagenicity of PhIP in the colon of male and female Big Blue rats and concluded that the MFs in the colon mucosa were enhanced by treatment with PhIP, but there were no gender differences in the MFs (Table 3). Masumura et al. [110] examined the organ specificity of PhIP-induced mutations in male and female *gpt* delta mice and reported that the highest MF was observed in the colon, followed by the spleen and liver. There were no gender differences in the MFs in the colon and liver. Stuart et al. [111] also examined the organ specificity of PhIP in Big Blue rats and reported that the MF in the colon was higher than that in the cecum and also that no gender differences were observed in the MFs in the colon.

**Table 3:** In vivo mutagenicity of heterocyclic amines in transgenic mice and rats

Chemical	Species	Gender	Administration	Selection	Organ	Dose	Judgement	Remarks	Reference
2-amino-1-methyl-6-phenylimidazo [4,5-*b*]pyridine (PhIP)	Muta Mouse, i.e., (BALB/c x DBA/2)CD2 F1 mice, but was heterozygous at the endogenous *Dlb-1* locus.	male and female	Diet for 30, 60 and 90 days and sacrificed between 1 and 3 weeks after the last treatments. Diet for 30 days at a dose of 250 ppm.	*lacI*	small intestine	100 ppm	+	Accumulation of mutations at both loci (*lacI* and *Dlb-1*) appears to be linear with both PhIP concentration and duration of exposure. PhIP was more mutagenic in the small intestine than in the colon.	[112]
						250 ppm	+		
						400 ppm	+		
					colon	100 ppm	+		
						250 ppm	±		
						400 ppm	+		
2-amino-1-methyl-6-phenylimidazo [4,5-*b*]pyridine (PhIP)	Muta Mouse = (BALB/c x DBA/2)CD2 F1 mice	male	Gavage for 4 days, and sacrificed 7 days later.	*lacZ*	large intestine	20 mg/kg/day	+	No mutagenicity was observed in any organs examined at doses of 2 and 0.2 mg/kg/day.	[113]
					small intestine	20 mg/kg/day	+		
					liver	20 mg/kg/day	+		
					kidney	20 mg/kg/day	–		
2-amino-1-methyl-6-phenylimidazo [4,5-*b*]pyridine (PhIP)	Muta Mouse	male	per os for 4 days and sacrified 7 days after the last treatment.	*lacZ*	intestine	20 mg/kg/day	+	About 2/3 (65%) of the induced mutations were base substitutions and about half were G:C to T:A transversions. DNA was obtained from Lynch et al. [113].	[114]
2-amino-1-methyl-6-phenylimidazo [4,5-*b*]pyridine (PhIP)	F344 rats	male and female	diet for 60 days	*lacI*	colon	400 ppm	+	G:C deletions including deletions at 5′-GGGA-3′ were the most frequent mutations. No significant difference was observed in MF between male and female.	[109]
2-amino-1-methyl-6-phenylimidazo [4,5-*b*]pyridine (PhIP)	(C57BL/6 x SWR)F1 mice	male and female	diet for 60 days	*lacI*	colon	400 ppm	+	G:C to T:A transversions in runs of guanine were the hot spot of base substitutions. DNA was obtained from Zhang et al. [112].	[115]
2-amino-1-methyl-6-phenylimidazo [4,5-*b*]pyridine (PhIP)	C57 BL6/J mice	male	diet for 13 weeks	*gpt* and Spi^−^	colon	400 ppm	+	The highest MF was observed in the colon followed by the spleen and the liver in both *gpt* and Spi^−^ selections. There were no gender differences in MF in the colon and the liver.	[110]
					spleen		+		
					liver		+		
					testis		–		
					brain		–		
					bone marrow		–		
2-amino-1-methyl-6-phenylimidazo [4,5-*b*]pyridine (PhIP)	(BBR x SD)F1 rats	female	gavage for 2 weeks (5 consequtive days per week)	*lacI*	Mammary duct	65 mg/kg/day	+	G:C to T:A transversions were the most frequent mutations, followed by G:C deletions including deletions at GGGA sequence.	[116]
2-amino-1-methyl-6-phenylimidazo[4,5-b]pyridine (PhIP)	C57 BL6/J mice	male	diet for 13 weeks	*gpt* and Spi^−^	colon	400 ppm	+	G:C to T:A transversions and single G:C deletions were the most frequently observed mutations by *gpt* and Spi^−^ selections, respectively. DNA was obtained from Masumura et al. [110].	[117]
2-amino-1-methyl-6-phenylimidazo [4,5-*b*]pyridine (PhIP)	F344 rats	male and female	diet for 60 days	*cII*	colon	400 ppm	+	Mutation spectra were similar between *cII* and *lacI* assays. G:C to T:A transversions were the most frequent mutations followed by G:C to C:G transversions, G:C to A:T transitions and G:C deletions.	[118]
2-amino-1-methyl-6-phenylimidazo [4,5-*b*]pyridine (PhIP)	F344 rats	male	diet for 61 days	*lacI*	prostate	200 ppm	+	The predominant mutation was G:C to T:A transversions and G:C deletions.	[119]
2-amino-1-methyl-6-phenylimidazo [4,5-*b*]pyridine (PhIP)	F344 rats	male and female	diet for 61 days	*lacI*	cecum	200 ppm	+	MF in the colon was higher than that in the cecum. G:C to T:A transversions were the most frequent mutations, followed by G:C to C:G transversions and G:C deletions. No differences in MF were observed between male and female in the colon although tumors were induced in the colon of male rats. Hormone may play a role in the induction of tumors in the colon of male rats.	[111]
					proximal colon	200 ppm	+		
					distal colon	200 ppm	+		
2-amino-1-methyl-6-phenylimidazo [4,5-*b*]pyridine (PhIP)	F344 rats	male	diet for 61 days	*lacI*	distal colon	200 ppm	+	The rats were fed a diet with conjugated linoleic acid (CLA, 0.5%, wt/wt) or 1,2-dithiole-3-thione (DTT, 0.005%, wt/wt) starting one week before PhIP treatments of 61 days. CLA and DTT significantly reduced PhIP-induced MF in the distal colon. In contrast, DTT significantly elevated MF in the cecum.	[120]
					cecum	200 ppm	+		
2-amino-1-methyl-6-phenylimidazo [4,5-*b*]pyridine (PhIP)	F344 rats	male and female	diet for 47 days	*lacI*	distal colon	100 ppm	+	The rats were fed a diet with conjugated linoleic acid (CLA, 1%, wt/wt) one week before starting PhIP treatments of 47 days. CLA reduced the MF in the distal colon but not in the cecum.	[121]
					cecum	100 ppm	+		
2-amino-1-methyl-6-phenylimidazo [4,5-*b*]pyridine (PhIP)	F344 rats	male and female	diet for 47 days	*lacI*	kidney	100 ppm	+	MF in male was significantly higher than that in female. Conjugated linoleic acid inhibited the mutation in female but not in male.	[122]
2-amino-1-methyl-6-phenylimidazo [4,5-*b*]pyridine (PhIP)	F344 rats	female	oral dosing for 12 days	*lacI*	mammary gland	75 mg/kg	+	Young (43-day-old) and aged (150-day-old) female rats exhibited similar PhIP-induced MFs in the mammary gland.	[123]
2-amino-1-methyl-6-phenylimidazo [4,5-*b*]pyridine (PhIP)	F344 rats	male and female	intraperitoneal injection	*lacI*	colon	100 mg/kg	+	Calory restriction with a 40%-reduced diet for 22 weeks did not affect PhIP-induced MF in the colon.	[124]
2-amino-1-methyl-6-phenylimidazo [4,5-*b*]pyridine (PhIP)	F344 rats	male	gavage for three times a week for 4 weeks or 8 weeks	*cII*	liver	70 mg/kg	–	MFs were significantly elevated by PhIP treatments in the colon, the spleen, the seminal vesicles and all lobes of the prostate. The MFs were higher in 8-week treatments than in 4-week treatments.	[125]
					kidney		–		
					ventral prostate		+		
					dorsolateral prostate		+		
					anterior prostate		+		
					seminal vesicle		+		
					colon		+		
					spleen		+		
2-amino-3-methylimidazo[4,5-*f*]quinoline (IQ)	C57Bl/*lacZ* (=C57Bl x Muta Mouse) and *c-myc*/*lacZ* (=C57Bl/6 J x CBA/J x Muta Mouse)	?	p.o. for 10 days and sacrificed 4 weeks after the dosing	*lacZ*	liver	20 mg/kg/day	+	c-*myc* appears to enhance IQ-induced MF in the liver.	[126]
2-amino-3-methylimidazo[4,5-*f*]quinoline (IQ)	F344 rats	male	gavate for a single day or for 5 consecutive days	*lacI*	liver	20 mg/kg/day	+	MF was highest in the liver, followed by the colon and the kidney. GC transversions in the liver and the colon and 1 bp G:C deletions in the liver and the kidney were induced. A single G deletion in the sequence 5′-CGGG was detected in the liver and the colon. Preferential sequences for base substitutions and deletions were 5′-CGC/T-3′ and 5′-CGGA-3′, respectively.	[127]
					colon	20 mg/kg/day	+		
					kineny	20 mg/kg/day	+		
2-amino-3-methylimidazo[4,5-*f*]quinoline (IQ)	F344 rats	male	diet for 3 weeks	*cII*	liver	20 mg/kg/day	–	IQ induced higher MF in the liver than in the colon.	[128]
						70 mg/kg/day	+		
						200 mg/kg/day	+		
					colon	20 mg/kg/day	–		
						70 mg/kg/day	+		
						200 mg/kg/day	+		
2-amino-3-methylimidazo[4,5-*f*]quinoline (IQ)	F344 rats	male	diet for 3 weeks	*lacI*	colon	70 ppm	+	The rats were fed a diet with IQ or IQ + sucrose (0, 3.45% or 13.4%) for 3 weeks. IQ and sucrose increased MF independently in the colon. There were no interactions between IQ and sucrose interms of induction of mutations.	[129]
2-amino-3-methylimidazo[4,5-*f*]quinoline (IQ)	S.D. rats	female	diet for 13 weeks	*gpt*	liver	300 ppm	+	G:C to T:A transversions and single guanine deletions were induced by IQ.	[130]
2-amino-3-methylimidazo[4,5-*f*]quinoline (IQ)	F344 rats	male	diet for 4 weeks	*gpt* and Spi^−^	liver	0.1 ppm	–	Spi^−^ selection and GST-P positive foci assay were positive only in 100 ppm, while *gpt* assay was positive in 10 and 100 ppm. Frequencies of G:C to T:A transversions in the *gpt* gene were significantly increased in 1, 10 and 100 ppm in a dose-dependent manner.	[131]
						1 ppm	–		
						10 ppm	+		
						100 ppm	+		
2-Amino-3,4-dimethylimidazo[4,5-*f*]quinoline (MeIQ)	C57BL/6 mice	female	diet for 12 weeks	*lacI*	liver	300 ppm	+	G:C to T:A transversions, followed by G:C to A:T transitions, were induced in the liver and the bone marrow. DNA was obrained from Suzuki et al. [132].	[133]
					bone marrow	300 ppm	+		
2-Amino-3,4-dimethylimidazo[4,5-*f*]quinoline (MeIQ)	C57BL/6 mice	female	diet for 1, 4, 12 weeks	*lacI*	bone marrow	300 ppm	+	MF was in the order of the colon, the bone marrow, the liver and the forestomach. The MF increased in a feeding period-dependent manner. No mutagenicity was observed in the heart.	[132]
					liver	300 ppm	+		
					forestomatch	300 ppm	+		
					colon	300 ppm	+		
					heart	300 ppm	–		
2-Amino-3,4-dimethylimidazo[4,5-*f*]quinoline (MeIQ)	C57BL/6 mice	female	diet for 84 days	*lacI*	colon	300 ppm	+	G:C to T:A transversions at 5′-GC-3′ were the hot spot of base substitutions. DNA was obtained from Suzuki et al. [132].	[115]
2-Amino-3,8-dimethylimidazo[4,5-*f*]quinoxaline (MeIQx)	C57Bl/*lacZ* (=C57Bl x Muta Mouse) and *c-myc*/*lacZ* (=C57Bl/6 J x CBA/J x Muta Mouse)	?	p.o. for 10 days, and sacrified 4 weeks after the dosing	*lacZ*	liver	20 mg/kg/day	+	c-myc appears to enhance MeIQx-induced MF in the liver.	[126]
2-amino-3,8-dimethylimidazo[4,5-*f*]quinoxaline (MeIQx)	C57Bl/*lacZ* (=C57Bl x Muta Mouse) and *c-myc*/*lacZ* (=C57Bl/6 J x CBA/J x Muta Mouse)	male and female	diet for 30 or 40 weeks	*lacZ*	liver	600 ppm	+	MFs in the mice fed diets containing MeIQx for 30 or 40 weeks were about 40 times higher than those of untreated mice. MF in male was higher than that in female. There was a synergistic effects of MeIQx and c-*myc* for hepatocarcinogenesis. c-*myc* also enhanced MeIQx-induced MF.	[134]
2-amino-3,8-dimethylimidazo[4,5-*f*]quinoxaline (MeIQx)	C57BL/6 mice	male	single intragastric administration	*lacI*	liver	100 mg/kg	–	No mutagenicity was observed by single intragastric administration. Mutagenicity was observed only in female by 4 weeks administration. Both sexes were positive in 12 weeks administration. MF was higher in female than in male mice, which is consistent with the sensitivity to carcinogenicity of MeIQx.	[135]
					colon	100 mg/kg	–		
		male and female	diet for 4 weeks	*lacI*	liver	300 ppm	+ female/− male		
					colon	300 ppm	+ female/− male		
			diet for 12 weeks	*lacI*	liver	300 ppm	+		
					colon	300 ppm	+		
2-amino-3,8-dimethylimidazo[4,5-*f*]quinoxaline (MeIQx)	C57 BL6/J mice	male	diet for 12 weeks	*gpt*	liver	3 ppm	–	The Liver was more sensitive to the mutagenicity of MeIQx than the colon. MF at 300 ppm for 78 weeks in liver was higher than that at the same dose for 12 weeks. Whole *gpt* MF and the frequencies of G:C to T:A were not significantly increased at 3 ppm.	[136]
						30 ppm	+		
						300 ppm	+		
			diet for 12 weeks	*gpt*	colon	3 ppm	–		
						30 ppm	–		
						300 ppm	+		
			diet for 78 weeks	*gpt*	liver	300 ppm	+		
2-amino-3,8-dimethylimidazo[4,5-*f*]quinoxaline (MeIQx)	F344 rats	male	diet for 16 weeks	*lacI*	liver	0.01 ppm	–	MF was increased at 10 and 100 ppm. GST-P positive foci were induced at 100 ppm only. Most frequently induced mutations were frameshifts in guanine bases, followed by G to T transversions.	[137]
						0.1 ppm	–		
						1 ppm	–		
						10 ppm	+		
						100 ppm	+		
2-amino-3,8-dimethylimidazo[4,5-*f*]quinoxaline (MeIQx)	B6C3F(1) p53 (+/+) or p53 (+/−) mice	female	diet for 13 weeks with or without CCl_4_ (i.p. 1 ml/kg once a week)	*gpt* and Spi^−^	liver	300 ppm	+	G:C to T:A transversions were induced by MeIQx. CCl_4_ treatments enhanced MF even in the p53-deficient background.	[138]
9-(4′-aminophenyl)-9*H*-pyrido[3,4-*b*]indole (aminophenylnorharman, APNH)	C57 BL6/J mice	male	diet for 12 weeks	*gpt* and Spi^−^	liver	10 ppm	+	G:C to T:A transversions, followed by G:C to A:T transitions, were the most frequent mutations detected by *gpt* assay. Single G deletions in run sequences were detected by Spi^−^ selection. The liver was more sensitive than the colon in terms of induction of mutations.	[139]
						20 ppm	+		
				*gpt*	colon	10 ppm	+		
						20 ppm	+		
5-amino-6-hydroxy-8*H*-benzo [6, 7]azepino[5,4,3-*de*]quinolin-7-one (ABAQ)	C57 BL6/J mice	male	gavege for 3 weeks (5 consequtive days per week)	*gpt* and Spi^−^	liver	25 mg/kg	+	G:C to A:T transitions and A:T to C:G transversions were increased in the liver. Both *gpt* and Spi^−^ assays were positive in the liver but negative in the kidney.	[140]
						50 mg/kg	+		
					kidney	25 mg/kg	–		
						50 mg/kg	–		
2-amino-9H-pyrido[2,3-*b*]indole (AαC)	C57Bl/*lacZ* (=C57Bl x Muta Mouse) and *c-myc*/*lacZ* (=C57Bl/6 J x CBA/J x Muta Mouse)	?	p.o. for 10 days, and sacrified 4 weeks after the dosing	*lacZ*	liver	20 mg/kg/day	+	c-*myc* appears to enhance MF. MF of AαC was lower than those of IQ and MeIQx although DNA adduct levels were higher in AαC than in IQ or MeIQx.	[126]
2-amino-9H-pyrido[2,3-*b*]indole (AαC)	Muta Mouse, i.e., (BALB/c x DBA/2)CD2 F1 mice, but was heterozygous at the endogenous *Dlb-1* locus.	male and female	diet for 30 and 45 days	*lacI*	small intestine	800 ppm	–	AαC was mutagenic in the colon but not in the small intestine.	[112]
					colon	800 ppm	+		
2-amino-9H-pyrido[2,3-*b*]indole (AαC)	(C57BL/6 x SWR) F1 mice	male and female	diet for 45 days	*lacI*	colon	800 ppm	+	G:C to T:A transversions at 5′-CGT-3′ was the hot spot of base substitutions. DNA was obtained from Zhang et al. [112].	[115]

IQ induces intestinal tumors and hepatocellular carcinoma but not in the kidney of rats [141–143]. Bol et al. [127] examined the mutagenicity of IQ in Big Blue rats and reported that the highest MF was observed in the liver, followed by the colon and kidney, a non-target organ. The higher MF in the liver than in the colon induced by IQ was also reported by Moller et al. [128]. MeIQ induces tumors in the Zymbal gland, oral cavity, colon, skin and mammary glands in F344 rats and tumors in the liver and forestomach of CDF1 mice [143]. Suzuki et al. [132] examined the mutagenicity of MeIQ in female Big Blue mice (C57BL/6N) and reported that the highest MF was in the colon, followed by the bone marrow, the liver and the forestomach. MeIQx induces liver tumors in CDF1 mice where the female mice are more susceptible than males, but does not induce tumors in the colon in both sexes [144]. Itoh et al. [135] examined the mutagenicity of MeIQx in Big Blue mice (C57BL/6) and reported that the MF in the liver was higher in female mice than in males. They also observed an increase in MFs in the colon, a non-target organ for carcinogenesis, where no obvious differences in MFs between male and female were observed. Mutagenicity in the colon of mice has also been reported in male *gpt* delta mice fed a diet containing MeIQx [136]. APNH is formed from aniline and norharman *in vitro* and *in vivo* and induces liver and colon cancers in F344 rats [145]. The *in vivo* mutagenicity of APNH was examined in male *gpt* delta mice fed a diet containing 10 or 20 ppm of APNH for 12 weeks [139]. The MF was higher in the liver than in the colon, and the MF in the liver of the mice at 20 ppm was almost equivalent to that of the liver in the same mice fed a diet containing 300 ppm MeIQx for 12 weeks [136]. ABAQ is a heterocyclic amine formed from glucose and L-tryptophan via the Maillard reaction. ABAQ has a tumor initiating-activity in the colon of mice [146]. The *in vivo* mutagenicity of ABAQ was examined in male *gpt* delta mice treated by gavage for 3 weeks at 25 or 50 mg/kg [140]. The MFs in the liver increased in a dose-dependent manner, and no MF was enhanced by the treatments in the kidney. AαC is the second most abundant heterocyclic amine in very well-done meat and fish [147]. It induces cancers in the liver and blood vessels of CDF1 mice [148]. The *in vivo* mutagenicity of AαC was examined in F1 (C57BL/6 x SWR) mice with *lacI* as a reporter gene [112]. AαC enhanced MFs in the colon but not in the small intestine.

#### Mutation spectrum

Mutagens induce specific types of sequence changes in the genome, such as T to C mutations by ethyl nitrosourea, G to T mutations by benzo[*a*]pyrene and CC to TT by ultraviolet light irradiation. DNA sequence changes associated with mutagenic treatments are called the “mutation spectrum”. In particular, specific sequence changes in cancer cells are called “mutational signatures,” which are important clues for investigating the causes of human cancer [149, 150]. PhIP induces colon cancer in male F344 rats where the *adenomatous polyposis coli* (*Apc*) gene is mutated by a guanine deletion at a 5’-GGGA-3’ [151]. Okonogi et al. [109] examined the mutation spectrum in the colon of Big Blue rats fed a diet containing PhIP and reported that one bp deletion was the most frequent mutation, including a guanine deletion at 5’-GGGA-3’ in male and female rats. Okochi et al. [116] investigated the mutation spectrum of mammary glands in female F1 (Big Blue rat x SD) rats administered 10 gavages of PhIP and concluded that G:C to T:A transversions were the most frequent mutations, followed by G:C deletions including G:C deletions at a 5’-GGGA-3’. Stuart et al. [119] examined the mutation spectrum in the prostate of Big Blue rats fed a diet containing PhIP and concluded that the predominant mutations were G:C to T:A transversions and deletions of G:C bp. In mice, Lynch et al. [114] treated Muta Mouse with PhIP and examined the mutation spectrum in the intestine. Approximately 40% of the total mutations were G:C to T:A transversions and 20% were G:C deletions, which were similar to those observed in the *Hprt* and *DHFR* genes in hamster and human cells *in vitro*. Okonogi et al. [115] examined the mutation spectrum of PhIP in the colon of Big Blue mice and reported that approximately half of the mutations were G:C to T:A transversions, in particular in runs of guanine, and approximately 1/4 of the total mutations were G:C deletions. In the colon, the rate of G:C to T:A transversions is significantly higher in mice than in rats [109]. Masumura et al. [117] treated male *gpt* delta mice with PhIP and reported that G:C to T:A transversions and G:C deletions in particular in 5’-TTTTTTG-3’ to 5’-TTTTTT-3’ were predominant mutations in the colon detected by *gpt* and Spi^-^ selections, respectively. Overall, it seems that PhIP induces G:C to T:A transversions and G:C deletions and that the transversions are more frequently induced in mice than in rats.

IQ predominantly induces G:C to T:A transversions in the liver of *gpt* delta rats and also in the liver and colon of Big Blue rats [127, 130]. G:C to T:A was also induced by MeIQ in the liver, bone marrow and colon of female Big Blue mice [115, 133], APNH in the liver and colon of male *gpt* delta mice [139] and AαC in the colon of Big Blue mice [115]. Mutational hot spots for G:C to T:A transversions by PhIP, MeIQ and AαC are in runs of guanine, at 5’-GC-3’ and in 5’-CGT-3’, respectively [115].

#### No-observed effect level (NOEL) of in vivo mutagenesis

Toxicological assays, including *in vivo* mutagenicity assays of chemicals, are conducted at high doses, i.e., the maximum tolerable doses (MTDs), which are often 1,000 or 10,000 times higher than the human exposure levels in daily life. Therefore, it is unclear whether the toxicity or mutagenicity observed at high doses can also be observed at low doses where humans are actually exposed to the chemical [152]. Lynch et al. [113] examined the mutagenicity of PhIP in Muta mice treated by oral gavage at doses of 0.2, 2 and 20 mg/kg for 4 days and reported that PhIP was mutagenic only at a dose of 20 mg/kg in the large intestine and liver. No mutagenicity was observed in the kidney, even at 20 mg/kg. They suggested that 2 mg/kg may be a potential threshold dose for PhIP-induced mutagenesis. They argued, however, that the dose may be a detection limit instead of a threshold because of the high spontaneous MFs in the liver of Muta mice. Gi et al. [131] examined the mutagenicity of IQ in male F344 *gpt* delta rats fed diets at doses of 0.1, 1, 10 or 100 ppm for 4 weeks and reported that *gpt* MFs were significantly enhanced over the control level at doses of 10 and 100 ppm but not at 0.1 and 1 ppm in the liver. They reported, however, that the frequencies of G:C to T:A transversions were significantly enhanced over the control level at a dose of 1 ppm in addition to 10 and 100 ppm and that the increase in the frequencies was dose-dependent. It suggests that DNA sequencing analysis may enhance the sensitivity of mutation detection, thereby lowering the no-observed-effect level (NOEL). Masumura et al. [136] examined the mutagenicity of MeIQx in male *gpt* delta mice fed diets containing MeIQx at doses of 3, 30 or 300 ppm for 12 weeks. The MFs in the liver significantly increased at doses of 30 and 300 ppm but not at 3 ppm. The frequency of G:C to T:A did not significantly increase at 3 ppm, either. In this case, DNA sequencing analysis did not affect the NOEL. Hoshi et al. [137] examined the mutagenicity of MeIQx in male F344 Big Blue rats fed diets at doses of 0.01, 0.1, 1, 10 or 100 ppm for 16 weeks and reported that the MFs significantly increased at 10 and 100 ppm in the liver. In addition, they examined glutathione S-transferase placental form (GST-P)-positive foci in the liver, which is a marker for hepatocarcinogenesis. The number of GST-P-positive foci significantly increased beyond the number of the control group only at a dose of 100 ppm. They suggested that the NOEL for *in vivo* mutagenesis was lower than that for carcinogenesis.

### Implication of in vitro and in vivo mutation assays

The discovery of carcinogenic heterocyclic amines is one of the most fruitful scientific achievements enabled by the Ames test. Before this test was developed, the identification of chemical carcinogens solely depends on time-consuming animal tests. Multiple validation studies with more than 2,000 chemicals revealed that approximately 70-90% of chemical carcinogens are positive in the Ames test [153]. Therefore, this test is adopted in OECD test guideline 471 [154] and is widely used to eliminate potential carcinogens from pre-marketing chemicals developing for pharmaceuticals, pesticides, food additives and others. Owing to the power of the Ames test, it was initially expected that strong mutagens in the Ames test might be strong carcinogens in rodents. However, studies with a large database indicated that the potency in the Ames test does not quantitatively correlate with that in rodent carcinogenicity assays [155]. The lack of quantitative relationships between mutagenesis in bacteria and carcinogenesis in rodents may not be very surprising when considering the complex process of carcinogenesis such as mutation or initiation, promotion and progression. Since *in vivo* mutagenesis is much simpler than carcinogenesis, it was expected that the potency of the Ames test might correlate with that in transgenic mutation assays quantitatively. Although the mutagenic potency (revertants per μg) of MeIQ in strain TA98 is more than 300 times higher than that of PhIP [6], the MF of MeIQ in the colon of Big Blue mice fed a diet containing 300 ppm for 90 days is similar to that of PhIP in the mice fed a diet containing 400 ppm for 90 days [156]. It seems, therefore, that the mutagenic potency of the Ames test does not quantitatively correlate with the potency in *in vivo* mutation assays. It is also pointed out that the potency of the Ames test does not quantitatively correlate with that in *in vitro* mammalian cell assays for gene mutation and chromosome aberrations [153]. Despite the lack of quantitative correlations, the power of the Ames test to qualitatively predict potential carcinogens is outstanding, as evidenced by the successful discovery of carcinogenic heterocyclic amines.

Transgenic rodent mutation assays have enabled us to analyze chemical-induced mutations in various organs and tissues at the sequence level. Therefore, it would be interesting to examine whether we can predict target organs and sensitive gender for carcinogenesis based on the high MFs in specific organs and gender of rats and mice. Thus, the MFs were compared between the target organs and non-target organs for carcinogenesis, and the gender specificity in mice and rats was examined. However, the MFs in various lobes of the prostate were almost equally sensitive to the mutagenicity of PhIP, while the ventral prostate was the only target for cancer induction in rats [108, 125]. MeIQ induces much higher MF in the colon than in the liver, but the cancer incidence is higher in the liver than in the colon in mice [132, 157]. PhIP induces mutations in the colon of male and female rats, while colon cancer is induced only in males [107–109, 111]. MeIQx induces mutations in the colon of male and female mice, but it does not induce tumors in the colon [135, 136, 144]. These results indicate that target organs or tissues for carcinogenesis do not necessarily exhibit higher MFs compared to other organs or tissues, and also that mutations can be induced regardless of the gender specificity for carcinogenesis. In other words, the organs or tissues that are positive in the transgenic mutation assays are not necessarily carcinogenic targets. It appears, however, that tumors are induced in organs where mutations are induced when the carcinogens are genotoxic. Therefore, the transgenic mutation assays are employed to distinguish genotoxic carcinogens from non-genotoxic carcinogens [158]. The results of the transgenic mutation assays reflect *in vivo* metabolism and mammalian DNA repair, while the results of the Ames test reflect *in vitro* metabolism of S9 and bacterial DNA repair. Hence, the *in vivo* mutation assays may be useful to narrow down genotoxic carcinogens from chemicals that are positive in the Ames test. In fact, International Conference on Harmonization of Technical Requirements for Registration of Pharmaceuticals for Human Use (ICH) M7 for regulation of mutagenic impurities in pharmaceuticals recommends conducting *in vivo* mutation assays when the chemical is mutagenic in the Ames test [159]. Research on carcinogenic heterocyclic amines has provided valuable lessons on the effectiveness and limitations of *in vivo* transgenic mutation assays.

Since carcinogenic heterocyclic amines are produced by cooking, a question is whether they induce cancers in humans. If so, the extent to which they impose cancer risks on the general population? The exposure levels of heterocyclic amines are reported to be less than 500 ng per person per day [6]. In general, genotoxic carcinogens are regulated under the policy that they have no threshold or safe doses [152, 159, 160]. Therefore, there is carcinogenic risk to people who take carcinogenic heterocyclic amines. However, humans have various protective mechanisms against mutagenic substances such as detoxification, DNA repair, error-free translesion synthesis and apoptosis [161]. It is expected, therefore, that low-dose exposure to mutagenic carcinogens may be negligible due to these mechanisms. In addition, people are constantly exposed to endogenous mutagens such as reactive oxygen species. Thus, mutagenic risk is inevitable in humans. European Food Safety Authority (EFSA) and World Health Organization (WHO) propose 150 ng per person per day as a sufficient protective threshold of toxicological concern (TTC) for DNA-reactive genotoxic chemicals [162, 163]. Several studies with transgenic rodents exposed to low levels of carcinogenic heterocyclic amines have suggested the presence of NOEL [113, 131, 136, 137]. Although TTC is a concept that was developed to prioritize chemicals that require further toxicological evaluation and NOEL does not mean the absolute safe level, there may be certain exposure levels for genotoxic carcinogens, which do not increase excess lifetime cancer risk substantially. However, humans are exposed to multiple chemicals. Therefore, the combined risk should be evaluated. It has been reported that six carcinogenic heterocyclic amines, each of whose doses was below non-detectable levels by the Ames test, became mutagenic when they were combined [164]. In addition, chemicals may exhibit co-mutagenic effects and produce mutagenic substances when more than one non-mutagenic substance is combined [165]. Risk assessment of multiple exposures to DNA reactive mutagenic carcinogens at low levels may be a challenge that research on carcinogenic heterocyclic amines has proposed us.

## Conclusions

*Salmonella typhimurium* YG strains help in the discovery of novel carcinogenic heterocyclic amines in complex mixtures such as food and river water by the Ames test because of the high sensitivity to mutagenic aromatic amines and nitroaromatics. Strain YG1024, which overproduces acetyltransferase, exhibited much higher sensitivity than TA98 for Glu-P-1, IQ, MeIQ, MeIQx and PBTA-1 but not for PhIP and Trp-P-2. It suggests that some of the heterocyclic amines are not activated by acetyltransferase. Transgenic rodent in vivo mutation assays are useful to analyze mutations in any organs of mice and rats at the sequence level. Heterocyclic amines induced tumors in the organs where mutations are induced. However, not all the organs where mutations are induced are target organs for carcinogenesis and the target organs for carcinogenesis are not necessarily organs where the highest MFs are observed. Research on carcinogenic heterocyclic amines provided valuable insights into the effectiveness and the limitation of in vitro and in vivo mutation assays for the identification of human carcinogens.

## Supplementary Information

**Additional file 1.**

## Data Availability

Not applicable.

## Abbreviations

IARC: International Agency for Research on Cancer
Trp-P-1: 3-amino-1,4-dimethyl-5*H*-pyrido [4,3-*b*]indole
Trp-P-2: 3-amino-1-methyl-5*H*-pyrido [4,3-*b*]indole
Glu-P-1: 2-amino-6-methyldipyrido [1,2-*a*,3′,2′-*d*]imidazole
Glu-P-2: 2-aminodipyrido [1,2-*a*:3′,2′-*d*]imidazole
AαC: 2-amino-9*H*-pyrido [2,3-*b*]indole
MeAαC: 2-amino-3-methyl-9*H*-pyrido [2,3-*b*]indole
*Salmonella typhimurium*: *Salmonella enterica* subsp. *enterica* serovar Typhimurium
IQ: 2-Amino-3-methylimidazo [4,5-*f*]quinoline
MeIQ: 2-amino-3,4-dimethylimidazo [4,5-*f*]quinoline
MeIQx: 2-amino-3,8-dimethylimidazo [4,5-*f*]quinoxaline
PhIP: 2-amino-1-methyl-6-phenylimidazo [4,5-*b*]pyridine
S9: 9000 x g supernatant of rat liver homogenates
S9 mix: S9 plus an NADPH-generating system
PBTA-1: 2-[2-(acetylamino)-4-[bis (2-methoxyethyl)amino]-5-methoxyphenyl]-5-amino-7-bromo-4-chloro-2*H*-benzotriazole
ABAQ: 5-amino-6-hydroxy-8*H*-benzo [1, 2] azepino [5,4,3-*de*]quinolin-7-one
APNH or aminophenylnorharman: 9-(4′-aminophenyl)-9*H*-pyrido [3,4-*b*]indole
N-OH-APNH or hydroxyaminophenylnorharman: 9-(4′-hydroxyaminophenyl)-9*H*-pyrido [3,4-*b*]indole
amino-3′-methylphenylnorharman: 9-(4′-amino-3′-methylphenyl)-9*H*-pyrido [3,4-*b*]indole
amino-2′-methylphenylnorharman: 9-(4′-amino-2′-methylphenyl)-9*H*-pyrido [3,4-*b*]indole
MF: mutant frequency
bps: base pairs
6-TG: 6-thioguanine
SD: Sprague Dawley
F344: Fischer 344
*Apc*: *adenomatous polyposis coli*
MTD: maximum tolerable dose
NOEL: no-observed effect level
GST-P: glutathione S-transferase placental form
ICH: International Conference on Harmonization of Technical Requirements for Registration of Pharmaceuticals for Human Use
EFSA: European Food Safety Authority
WHO: World Health Organization
TTC: threshold of toxicological concern
