# Supplementary material for: Mutagenicity of carcinogenic heterocyclic amines in Salmonella typhimurium YG strains and transgenic rodents including gpt delta
Source: Genes Environ. 2021 Sep 16;43:38. doi: 10.1186/s41021-021-00207-0 (PMC8444484; doi:10.1186/s41021-021-00207-0)
Supplement: Supplementary file 1 — Additional file 1. [file 41021_2021_207_MOESM1_ESM.pdf]

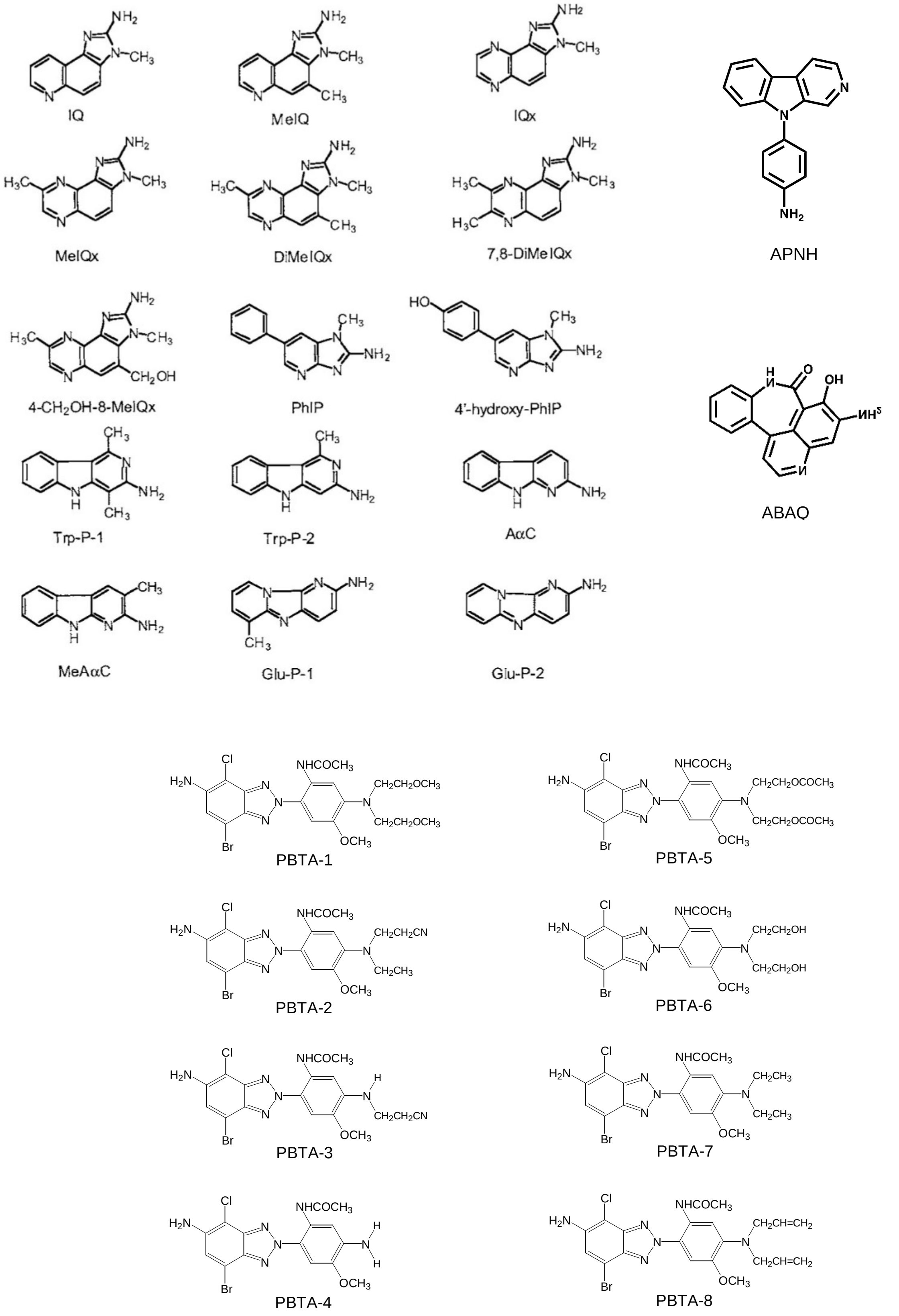

Supplementary Fig. 1 Structures of heterocyclic amines

The structural formula of IQ, MeIQ, IQx, MeIQx, DiMeIQx, 7,8-DiMeIQx, 4-CH_2OH-8-MeIQx, PhIP, 4'-hydroxy-PhIP, Trp-P-1, Trp-P-2, AαC, Me AαC, Glu-P-1, Glu-P-2 were cited from reference 6 with permission.

The structural formula of APNH and ABAQ were kindly provided by Professor Yukari Totsuka, School of Pharmacy, Nihon University, Chiba, Japan. The structural formula of PBTAs were kindly provided by Professor Tetsushi Watanabe, Department of Public Health, Kyoto Pharmaceutical University, Kyoto, Japan.

Supplementary Table 1 Chemical name and CAS number of heterocyclic amines

Common abbreviation	CAS number	Full name
IQ	76180-96-6	2-Amino-3-methylimidazo[4,5-*f*]quinoline
MeIQ	77094-11-2	2-amino-3,4-dimethylimidazo[4,5-*f*]quinoline
IQx	108354-47-8	2-amino-3-methylimidazo[4,5-*f*]quinoxaline
MeIQx	77500-04-0	2-amino-3,8-dimethylimidazo[4,5-*f*]quinoxaline
DiMeIQx	95896-78-9	2-amino-3,4,8-trimethylimidazo[4,5-*f*]quinoxaline
7,8-DiMeIQx	92180-79-5	2-amino-3,7,8-trimethylimidazo[4,5-*f*]quinoxaline
4-CH_2OH-8-MeIQx	153954-29-1	2-amino-4-hydroxymethyl-3,8-drimethylimidazo[4,5-*f*]quinoxaline
PhIP	105650-23-5	2-amino-1-methyl-6-phenylimidazo[4,5-*b*]pyridine
4'-hydroxy-PhIP	126861-72-1	2-amino-6-(4-hydroxyphenyl)-1-methylimidazo[4,5-*b*]pyridine
Trp-P-1	62450-06-0	3-Amino-1,4-dimethyl-5*H*-pyrido[4,3-*b*]indole
Trp-P-2	62450-07-1	3-amino-1-methyl-5*H*-pyrido[4,3-*b*]indole
AαC	26148-68-5	2-amino-9*H*-pyrido[2,3-*b*]indole
Me AαC	68006-83-7	2-amino-3-methyl-9*H*-pyrido[2,3-*b*]indole
Glu-P-1	67730-11-4	2-amino-6-methyldipyrido[1,2-*a*:3',2'-*d*]imidazole
Glu-P-2	67730-10-3	2-aminodipyrido[1,2-*a*:3',2'-*d*]imidazole
APNH	219959-86-1	9-(4'-aminophenyl)-9*H*-pyrido[3,4-*b*]indole
ABAQ		5-amino-6-hydroxy-8*H*-benzo[6,7]azepino[5,4,3-*de*]quinolin-7-one
PBTA-1	194590-84-6	2-[2-(acetylamino)-4-[bis(2-methoxyethyl)amino]-5-methoxyphenyl]-5-amino-7-bromo-4-chloro-2*H*-benzotriazole
PBTA-2	215245-16-2	2-[2-(acetylamino)-4-[*N*-(2-cyanoethyl)ethylamino]-5-methoxyphenyl]-5-amino-7-bromo-4-chloro-2*H*-benzotriazole
PBTA-3	270925-57-0	2-[2-(acetylamino)-4-[(2-hydroxyethyl)amino]-5-methoxyphenyl]-5-amino-7-bromo-4-chloro-2*H*-benzotriazole
PBTA-4	351995-07-8	2-[2-(acetylamino)-4-amino-5-methoxyphenyl]-5-amino-7-bromo-4-chloro-2*H*-benzotriazole
PBTA-5	392274-03-2	2-[4-[bis(2-acetoxyethyl)amino]-2-(acetylamino)-5-methoxyphenyl]-5-amino-7-bromo-4-chloro-2*H*-benzotriazole
PBTA-6	392274-07-6	2-[2-(acetylamino)-4-[bis-(2-hydroxyethyl)amino]-5-methoxyphenyl]-5-amino-7-bromo-4-chloro-2*H*-benzotriazole

PBTA-7	468062-49-9	2-[2-(acetylamino)-4-(diethylamino)-5-methoxyphenyl]-5-amino-7-bromo-4-chloro-2*H*-benzotriazole
PBTA-8	468062-50-2	2-[2-(acetylamino)-4-(diallylamino)-5-methoxyphenyl]-5-amino-7-bromo-4-chloro-2*H*-benzotriazole
